# Dpr10 and Nocte are required for *Drosophila* motor axon pathfinding

**DOI:** 10.1186/s13064-022-00165-5

**Published:** 2022-10-21

**Authors:** Meike Lobb-Rabe, Katherine DeLong, Rio J. Salazar, Ruiling Zhang, Yupu Wang, Robert A. Carrillo

**Affiliations:** 1 Department of Molecular Genetics & Cellular Biology, University of Chicago, Chicago, IL 60637 USA; 2 Neuroscience Institute, University of Chicago, Chicago, IL 60637 USA; 3 Program in Cell and Molecular Biology, University of Chicago, Chicago, IL 60637 USA; 4 Committee on Development, Regeneration, and Stem Cell Biology, University of Chicago, Chicago, IL 60637 USA

## Abstract

The paths axons travel to reach their targets and the subsequent synaptic connections they form are highly stereotyped. How cell surface proteins (CSPs) mediate these processes is not completely understood. The *Drosophila* neuromuscular junction (NMJ) is an ideal system to study how pathfinding and target specificity are accomplished, as the axon trajectories and innervation patterns are known and easily visualized. Dpr10 is a CSP required for synaptic partner choice in the neuromuscular and visual circuits and for axon pathfinding in olfactory neuron organization. In this study, we show that Dpr10 is also required for motor axon pathfinding. To uncover how Dpr10 mediates this process, we used immunoprecipitation followed by mass spectrometry to identify Dpr10 associated proteins. One of these, Nocte, is an unstructured, intracellular protein implicated in circadian rhythm entrainment. We mapped *nocte* expression in larvae and found it widely expressed in neurons, muscles, and glia. Cell-specific knockdown suggests *nocte* is required presynaptically to mediate motor axon pathfinding. Additionally, we found that *nocte* and *dpr10* genetically interact to control NMJ assembly, suggesting that they function in the same molecular pathway. Overall, these data reveal novel roles for Dpr10 and its newly identified interactor, Nocte, in motor axon pathfinding and provide insight into how CSPs regulate circuit assembly.

**Supplementary Information:**

The online version contains supplementary material available at 10.1186/s13064-022-00165-5.

## Introduction

Assembling a functional neural circuit requires several steps: neurogenesis, axon pathfinding, synaptogenesis, and subsequent maintenance. The *Drosophila* embryonic/larval neuromuscular junction (NMJ) is an excellent model to study these processes due to its relatively simple circuit architecture, its genetic accessibility, and the fact that many of the processes and molecules are conserved in vertebrates.

To assemble circuits, growth cones—specialized structures at the ends of developing axons—travel through dense extra-cellular milieus and use molecular signals to guide them to their final destinations. This sensing is mediated by cell surface proteins (CSPs) on the growth cone. Many proteins have been implicated in axon pathfinding, and they function as long- and short-range signals detected by cell surface receptors on the filopodia of the highly dynamic growth cone [1, 54]. Some of the best studied signals include the guidance cue Netrin-1, which signals through its receptors Frazzled and UNC-5 [9, 29] and the Robo-Slit pathway that drives axon pathfinding through repulsion in the developing nervous system [7, 17, 44]. Although these pathways are well characterized, they represent only a small fraction of the cues required to wire an entire nervous system [30, 54], and we still do not fully understand the entire repertoire of complex signaling molecules that mediate axon pathfinding.

The immunoglobulin superfamily (IgSF) is a large family of soluble and membrane bound proteins implicated in all stages of circuit assembly and axon pathfinding [37, 48]. An in vitro screen seeking to deorphanize ligand-receptor pairs revealed interactions between two *Drosophila* IgSF subfamilies: the Dprs and DIPs [43]. These IgSF CSPs interact heterophilically and some members also homodimerize; moreover, the unrelated Ig protein, Klingon, and a leucine-rich repeat (LRR) protein, cDIP, interact with a subset of Dprs and DIPs [43]. Dprs and DIPs are expressed throughout the nervous system and their functions are only beginning to be elucidated. Work from our lab and others have demonstrated Dpr-DIP function in cell survival [10, 61], synaptic partner preference [60], cell fate determination [16], and axon guidance in olfactory neurons [6]. Moreover, Dprs and DIPs are required for synaptic partner recognition in the adult brain [8, 10, 16, 38, 61] and in the NMJ [5, 55], suggesting multifaceted roles for Dprs and DIPs in nervous system development.

**Fig. 1:**
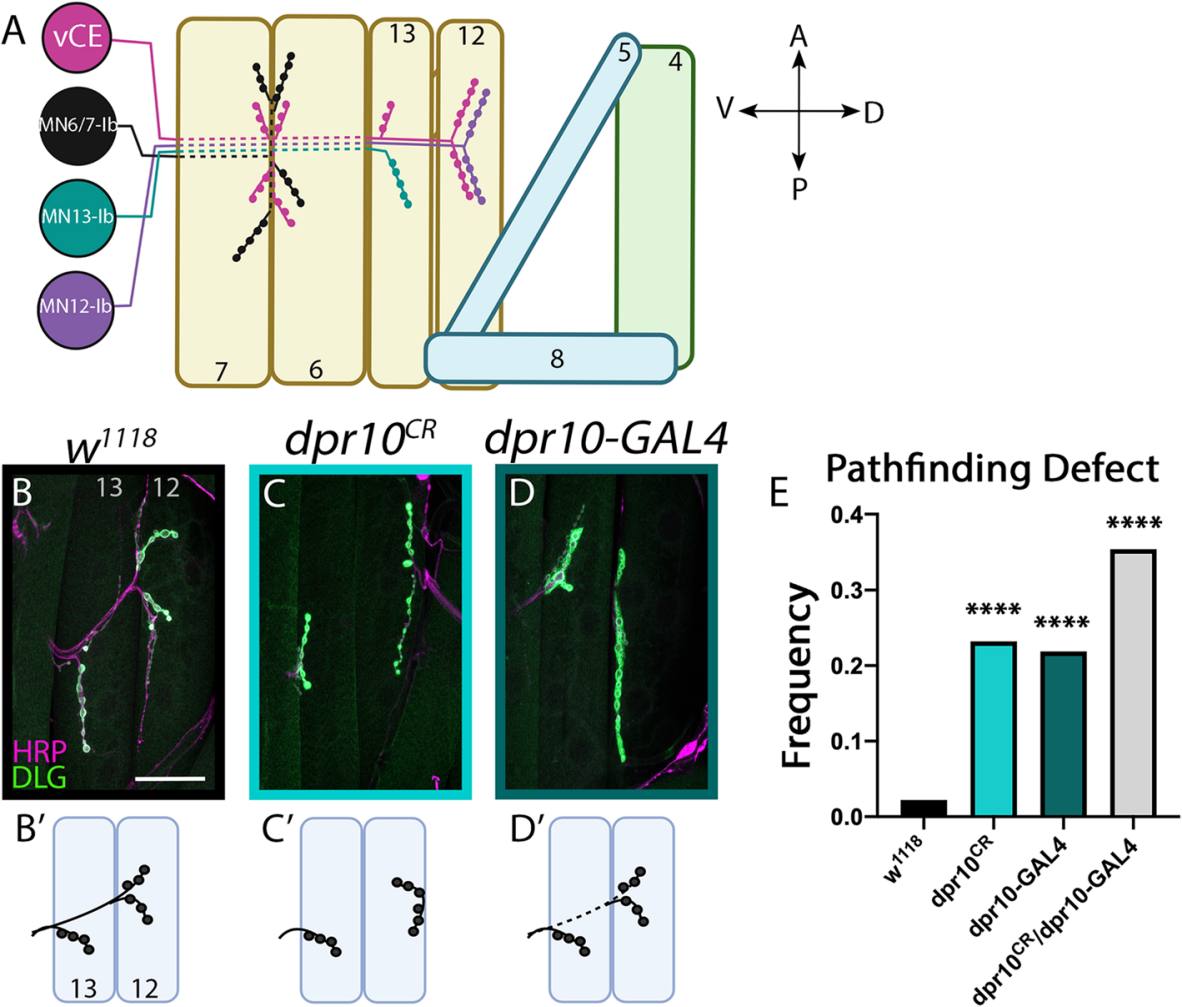
Dpr10 is required for ISNb pathfinding. (**A**) Schematic of innervation pattern of the ventral muscle field. The Is motor neuron, vCE, is shown in magenta and Ib neurons are shown in black, teal, and purple. Solid lines indicate the nerve is traveling above muscles while dotted line indicates the nerve is travelling below muscles. (**B**-**D′**) Innervation of m13 and m12 with neurons labeled by HRP staining (magenta) and postsynapses labeled with DLG staining (green). Scale bar = 50 μm. (**B,B′**) Control *w*^*1118*^ animal with the normal innervation pattern (*n* = 90). (**C,C′**) *dpr10*^*CR*^ animals (light blue) display significant ISNb pathfinding defects (*n* = 55). Here, m12 is innervated from distal side. (**D,D′**) *dpr10-GAL4* animals (dark green) display significant ISNb pathfinding defects (*n* = 64). Here, the nerve travels underneath m13 to innervate m12. (**E**) Quantification of ISNb pathfinding defects of control and *dpr10* mutant animals. *dpr10*^*CR*^*/dpr10-GAL4* mutants also displayed pathfinding defects, gray bar (*n* = 48). ****p < 0.0001

The larval NMJ is divided into highly stereotyped, segmentally repeated hemisegment units, and each hemisegment is comprised of 30 body wall muscles that are innervated by ~ 33 motor neurons. Most muscles are innervated by two glutamatergic motor neurons, the Ib (big) and Is (small) types, which can be distinguished by the size of their postsynaptic membrane architecture as visualized with staining for Discs Large (DLG) [22]. Most Ib motor neurons innervate single muscle targets, while Is motor neurons innervate groups of muscles. In this study, we examined the intersegmental nerve b (ISNb) consisting of efferent motor neuron axons that innervate a subset of the ventral muscle field (Fig. 1A) and afferent sensory neuron axons that project into the ventral nerve cord (VNC).

Dpr10 is required for olfactory neuron pathfinding but whether it also functions in motor neuron axon guidance is unknown. Here, we demonstrate that Dpr10 is required for ISNb pathfinding; loss of *dpr10* leads to misrouting of several motor axons in the ISNb, including those innervating muscles 12 and 13 (m12 and m13; also referred to as VL1 and VL2, respectively). To determine how Dpr10 mediates ISNb pathfinding, we performed Dpr10 immunoprecipitation-mass spectrometry from *Drosophila* larvae. Several proteins were identified, including cDIP, a previously identified Dpr10 interactor [10, 43], and the cytosolic protein Nocte.

To begin to uncover the role of Nocte in the neuromuscular system, we examined *nocte* expression in larvae and found it in motor neurons, muscles, and glia. Anatomical analyses of *nocte* mutants revealed ISNb pathfinding defects, and cell-specific knockdown demonstrated that Nocte is required in motor neurons for proper ISNb pathfinding. Genetic analyses revealed that these *nocte* phenotypes were shared with *dpr10* mutants, and that they likely act in the same pathway. Overall, our work implicates a Dpr10–Nocte pathway in motor axon pathfinding and demonstrates that Dpr10 is required in several steps of circuit assembly, including pathfinding and synaptic partner matching.

## Methods

### *Drosophila melanogaster* stocks and reagents

All flies were maintained at 25 °C. Crosses were kept at medium density and vials were flipped every day to achieve a standard level of egg laying and rearing conditions.

The Stanewsky Lab (University of Münster) generously provided all Nocte fly reagents (Table 1).

**Table 1:** *Drosophila* lines used in this study

Genotype	Description	Source
*nocte*^*P*^	Mutant (transposon excision)	[50]
*nocte*^*1*^*/ FM7*	Mutant (EMS)	[50]
*UAS-nocte-RNAi 2:1b;UAS-nocte-RNAi 1:3*	RNAi	[50]
*nocte-Gal4*	Nocte GAL4 driver	[50]
*UAS-Flag-Strep-nocte-HA*	FLAG and HA tagged nocte	[13]
*dpr10*^*CR*^*(also known as dpr10*^*null*^*and dpr10*^*14-5*^*)*	Mutant (CRISPR)	[5, 61]
*Mef2-GAL4*	Muscle GAL4 driver	[47]
*Elav-GAL4*	Neuronal GAL4 driver	BDSC: 8765
*Repo-GAL4*	Glial GAL4 driver	BDSC: 7415
*dpr10-T2A-GAL4*	Dpr10 MiMIC GAL4 insertion	[35]
*UAS-GFP::LacZ NLS*	Nuclear localized GFP	BDSC: 6451
*W*^*1118*^	White control	BDSC
*UAS-nRedStinger, UAS-FLP, Ubi-p63E(FRT.STOP)-nStinger*	G-TRACE	BDSC: 28280
*UAS-mCD8::GFP*	Membrane GFP	BDSC: 32184
*UAS-Dpr10-V5*	V5 tagged Dpr10	[61]

### Larval dissections

Larval dissections and immunostaining were performed as described previously [5]. Briefly, third instar larvae were dissected in PBS on Sylgard dishes and fixed with 4% paraformaldehyde for 30 minutes. Fillets were washed with Phosphate Buffered Saline (PBS) and placed in tubes with PBST (PBS + 0.05% TritonX100) for three 15-minute washes on a nutator, before blocking for an hour in 5% Normal Goat Serum in 0.05% PBST block. Samples were incubated with primary antibodies overnight in block (5% Goat serum in 0.05% PBST) at 4 °C. After incubation, samples were washed in PBST and incubated for 2 h with secondary fluorescent antibodies in block at room temperature. Samples were washed and then mounted in Vectashield (Vector Laboratories). Representative images were acquired on a Zeiss LSM800 confocal microscope and processed with ImageJ.

### Biochemistry

Fifty brains per sample were collected from *dpr10 > Dpr10-V5* and *dpr10 > CD8-GFP* larvae. Similarly, 50 body walls were collected from *Mef2 > Dpr10-V5* and *Mef2 > CD8-GFP* larvae. All dissections were performed in ice cold PBS and tissue was kept on ice for no more than 10 min before transferring to the − 80 °C freezer. Samples were homogenized in NP-40 Buffer with Pierce Protease Inhibitor Mini (A32955) using Fisherbrand RNase-FREE Disposable Pellet Pestles (12-141-368).

Tissue lysate was first cleared with Protein G Magnetic Beads (New England Bio Labs S1430S) and then immunoprecipitation was performed using fresh beads incubated with either mouse anti-V5 or mouse anti-GFP antibodies (1:8 antibody to bead ratio per sample). Samples were thoroughly washed before boiling in 2x SDS loading buffer. Samples were then run in a 12% acrylamide gel, stained with Coomassie Blue, and fragments were excised and placed in Eppendorf Tubes. Samples were digested with trypsin and liquid chromatography-mass spectrometry, and proteomics services were performed by the Northwestern Proteomics Core Facility, generously supported by NCI CCSG P30 CA060553 awarded to the Robert H. Lurie Comprehensive Cancer Center, instrumentation award (S10OD025194) from NIH Office of Director, and the National Resource for Translational and Developmental Proteomics supported by P41 GM108569. The mass spectrometry data was searched against a *Drosophila melanogaster* database and visualized with Scaffold Viewer 4 (Proteome Software).

### Embryos

Embryo dissections were performed as outlined by [34]. Briefly, embryo cages were setup in collection chambers, with grape plates at the bottom for egg laying. Each batch of grape plates is made by dissolving 12 g agar in 300 ml water and adding 100 ml grape juice, 5.35 g sucrose, and 8 ml Tegosept (Genesee Scientific 20–258). Yeast paste (dry yeast, water) was added to the center of grape plates to encourage egg laying and to feed adult flies. Plates were collected after a two-hour egg laying period at 25 °C in darkness. Embryos reached stage 16 after incubation in humid environment at 25 °C for 15 hours. Staging was confirmed by shape of the gut after chorion was removed using a dull metal probe and two-sided tape [34]. Embryos were staged on a slab of agar before being transferred, dorsal side up, to two-sided tape on a glass slide. Embryos were submerged in filter sterilized PBS. Using a sharp tungsten probe, embryos were removed from vitellin membrane and placed onto Superfrost Plus Slides. Embryos were fillet, fixed, and stained, similar to the larval protocol above, the only difference is that primary and secondary antibodies are incubated overnight at 4 °C and that samples were not placed on a nutator at any point.

For embryo hatching experiments, *w*^*1118*^ and *dpr10*^*CR*^ flies were transferred to cages and allowed to lay for 24 hours. Embryos were transferred to a fresh grape plate in a humidified chamber and incubated for 48 hours at 25 °C. Finally, the number of hatched embryos were counted, and the percentage of hatched embryos was calculated.

### Immunohistochemistry

The primary antibodies used for this study are: Chicken anti-GFP (1:500, Invitrogen A10262), Rabbit anti-GFP (1:10,000, Glotzer Lab), Chicken anti-mCherry (1:1000, Novus NBP 2–25,158), Mouse anti-HA (1:1000, BioLegend 901,501), Rabbit anti-HA (1:300, Cell Signaling 3724S), Rabbit anti-FLAG (1:200, Novus NBP-1-06712), Rat anti-FLAG (1:200, Novus Biologicals), Mouse anti-Repo (1:100, DSHB 8D12), Rabbit anti-DLG (1:40,000, Budnik Lab), Mouse anti-LamC (1:100, DSHB LC28.26), Mouse anti-V5 (1:200, Invitrogen R960–25), Mouse anti-Cut (1:100, DSHB LC28.26), Rabbit anti-pmad (1:300, Abcam EP823Y), Mouse anti-Eve (1:100, DSHB 3C10), and Mouse anti-FasII (1:100, DSHB 1D4).

The conjugated primary antibodies used for this study are: Goat anti-Phalloidin 405 (1:100, Invitrogen A30104), Goat anti-HRP 405 (1:100, Jackson ImmunoResearch 123–475-021), Goat anti-HRP TRITC (1:100, Jackson ImmunoResearch 123–025-021), and Goat anti-HRP 647 (Jackson ImmunoResearch 123-605-021).

The secondary antibodies used for this study are: Goat anti-Mouse 488 (1:500, Invitrogen A11029), Goat anti-Rabbit 488 (1:500, Invitrogen A11008), Goat anti-Chicken 488 (1:500, Invitrogen A11039), Goat anti-Rat 488 (1:500, Invitrogen A11006), Goat anti-Rat 568 (1:500, Invitrogen A11077), Goat anti-Mouse 568 (1:500, Invitrogen A11031), Goat anti-Rabbit 568 (1:500, Invitrogen A11036), Goat anti-Chicken Cy3 (1:500, Jackson Immunological Research 123-605-021), Goat anti-Rabbit 647 (1:500, Invitrogen A32733), and Goat anti-Mouse 647 (1:500, Invitrogen A32728).

### Imaging protocols

Quantification of innervation and misrouting phenotypes were conducted using a Zeiss Axiolmager M2 and a 40X plan-neofluar 1.3NA objective. NMJs were examined using HRP and DLG stains. The DLG signal allowed for scoring the presence of Is NMJs, as Is boutons have smaller postsynaptic membrane structures and appear smaller and dimmer than Ib boutons. All images were captured on a Zeiss LSM800 confocal microscope with a 20X plan-apo 0.8NA objective, a 40X plan-neofluar 1.3NA objective, or 63X plan-apo 1.4NA objective.

### Analysis

Statistical analysis was performed using GraphPad (Prism). Each data group was collected from a minimum of two experiments of six animals each, using the abdominal body wall segments 2–5. Data significance was determined through Chi Squared Test with Fisher’s exact test. Images were prepared using ImageJ FIJI [49].

## Results

### Dpr10 is required for ISNb pathfinding and innervation

Dpr10 is a member of the *Drosophila* IgSF and instructs synaptic partner choice in the pupal neuromuscular junction [55] and innervation of specific medulla layers in the pupal visual circuit [61]. In the olfactory bulb, Dpr10 is required for axon pathfinding of olfactory neurons [6]. Additionally, in the embryonic neuromuscular junction, Dpr10 is required for innervation of muscle 4 (m4) by the dorsal Is motor neuron (known as the dorsal common exciter, dCE, and RP2) [5]. *dpr10* is expressed in a large subset of embryonic and larval muscles and motor neurons [5, 10, 56], suggesting that it may have additional functions in neuromuscular junction development.

Each hemisegment of the *Drosophila* larval body wall is innervated by three nerves: the intersegmental nerve (ISN), segmental nerve (SN), and transverse nerve (TN). The ISN and SN are further divided based on the muscle groups they innervate. For example, motor neuron axons in the ISNb innervate a subset of the ventral muscles. For this study, we focused on a subset of ventral muscles: m13 and m12 (Fig. 1A). These muscles are innervated by a single Is motor neuron, the ventral common exciter (vCE or RP5, Fig. 1A) and two Ib motor neurons, MN13-Ib, and MN12-Ib, respectively. The routes that these motor axons travel to reach their muscle targets and the innervation patterns are highly stereotyped. For example, in a dissected larva, the ISNb exits the VNC at specific locations, follows a defined trajectory under m6 and m7, emerges above m13, and finally reaches the ventral side of m12. In control animals, this pattern is extremely hardwired with 97.8% of hemisegments revealing the correct ISNb trajectory (*n* = 90) (Fig. 1B). We scored ISNb pathfinding defects at m13 and m12 including motor axons aberrantly traveling under m13, under m12, or under both, and loss of innervation of any of the Is or Ib terminals on m13 or m12. These phenotypes were collectively reported as a ‘pathfinding defect’ as pathfinding can be aberrant by the path the axon takes and subsequently which muscles it innervates. Additional misrouting phenotypes were observed at other NMJs, but in this study we focus specifically on the ISNb at m12 and m13.

The expression of *dpr10* in embryonic and larval muscles and motor neurons prompted us to examine additional roles for Dpr10 in NMJ assembly. We used two *dpr10* alleles—a CRISPR generated null allele (*dpr10*^*CR*^) [61] and a T2A-GAL4 converted from a MiMIC insertion that contains transcriptional and translational stops (*dpr10-GAL4*) [35]. RT-qPCR from *dpr10-GAL4* larvae revealed that *dpr10* mRNA levels were significantly reduced to approximately 6% relative to controls, suggesting the allele is a severe hypomorph [56]. To determine if Dpr10 is required for motor axon pathfinding, we first examined ISNb pathfinding defects in the *dpr10*^*CR*^ null background. Loss of *dpr10* revealed a significant ten-fold increase in ISNb pathfinding errors relative to controls (23.2% defect, *n* = 55, *p* < 0.0001) including the nerve traveling under m12 to innervate the incorrect dorsal side or missing innervations of Is and/or Ib motor neurons. To confirm that the phenotype was due to loss of *dpr10* and not a second site mutation, we examined the *dpr10-GAL4* hypomorph and observed comparable pathfinding defects (21.9% defect, *n* = 64, *p* < 0.0001) (Fig. 1C-E). We also examined *dpr10*^*CR*^*/dpr10-GAL4* larvae and found comparable levels of pathfinding defects (35.4% defect, *n* = 48, *p* < 0.0001) (Fig. 1E). The penetrance of these misrouting phenotypes is similar to other studies that examined ISNb pathfinding at m12 and m13. For example, mutations in *capricious* and *tartan* result in misrouting in 15 and 40% of hemisegments, respectively [32]. In addition, loss of *dpr10* may affect viability and impede observation of more severe phenotypes. We examined embryo hatching and indeed, significantly fewer *dpr10*^*CR*^ embryos hatched compared to controls, suggesting that defects in *dpr10* null animals affect the success rate of embryo hatching (Supplementary Fig. 1A).

Finally, *dpr10* mutants also exhibited a variety of muscle patterning defects, including missing muscles, muscle duplication, and incorrect attachment sites (Supplementary Fig. 1B-D). Altered muscle patterning can affect motor neuron pathfinding and innervation [11, 12] so only hemisegments with normal muscle patterns were quantified.

### Dpr10 interacts with Nocte and cDIP in vivo

Previous in vitro studies determined that Dpr10 binds four Ig proteins: DIP-α, DIP-β, DIP-λ, and Klingon, and one LRR protein, cDIP [10, 15, 43]. However, in vivo interacting partners of Dpr10 and putative downstream signaling pathways have not been thoroughly examined. To identify direct and indirect interactors in an unbiased manner, we performed immunoprecipitation of a tagged Dpr10, followed by mass spectrometry in order to identify putative components of a Dpr10 signaling pathway.

**Fig. 2:**
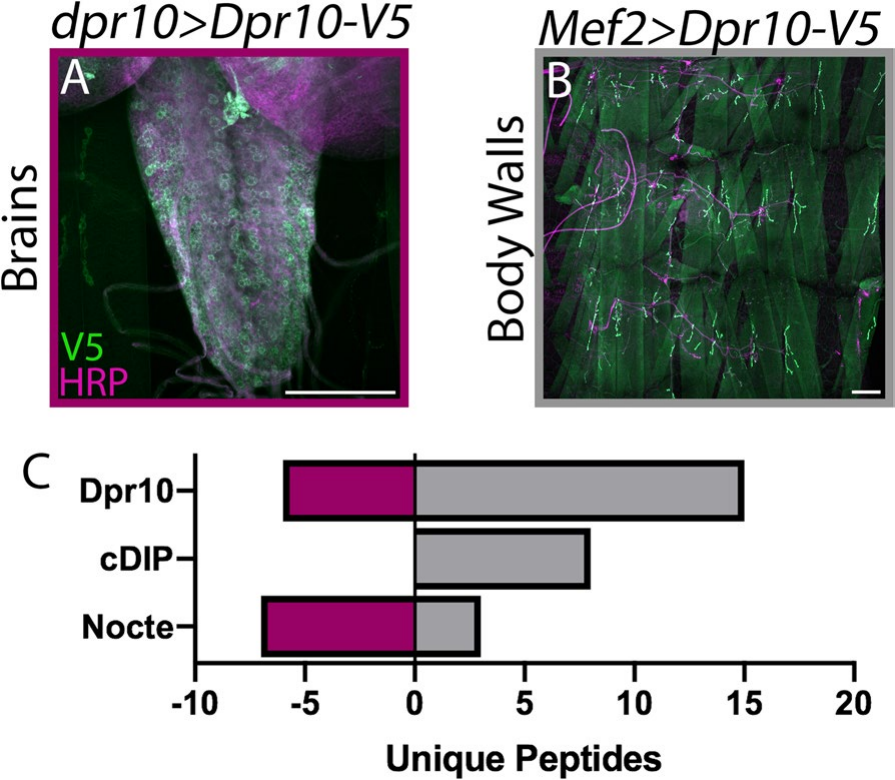
Immunoprecipitation followed by Mass Spectrometry of Dpr10 uncovers in vivo interactors. (**A**) Larval brain of Dpr10-V5 expressed with *dpr10-GAL4*. Preps were stained for V5 (green) to visualize Dpr10 and HRP (magenta) to visualize neurons. (**B**) Larval body wall of Dpr10-V5 expressed in muscles (*Mef2-GAL4*). Scale bars = 100 μm. (**C**) Unique peptides recovered from immunoprecipitation of Dpr10-V5 and analyzed by mass spectrometry from brain samples (left, plum) and muscles (right, gray). Note that Nocte is the only associated protein recovered from both samples. Control immunoprecipitations did not have peptides for any of these proteins

Dpr10 is expressed in a subset of larval neurons and muscles [10, 56]. We used the GAL4-UAS system to drive expression of a V5 tagged Dpr10 (Dpr10-V5) with *dpr10-GAL4* and isolated larval brains; this preparation reveals Dpr10 interactors in the cell bodies, axons, and dendrites of motorneurons and interneurons (Fig. 2A). In addition, we expressed Dpr10-V5 in muscles (*Mef2-GAL4*) and collected larval body walls; this preparation reveals Dpr10 interactors in muscles (Fig. 2B). For both experiments, we ran complimentary controls by expressing mCD8-GFP with the same drivers and continuing with the same experimental pipeline. Here, we only report Dpr10 interacting proteins that appeared in the experimental conditions and had no detectable peptides in the controls.

In the body wall preparations, mass spectrometry analysis revealed cDIP as a Dpr10 interactor, confirming the previous in vitro Dpr10–cDIP binding (Fig. 2C) [43]. Additionally, we identified a novel interactor, Nocte (Fig. 2C). Strikingly, Nocte was the only protein pulled down with Dpr10 in both brain and body wall preparations (Fig. 2C). Absent from our immunoprecipitation experiments were other known binding partners of Dpr10: DIP-α, DIP-β, and DIP-λ. One possibility is that these DIPs are part of an insoluble membrane fraction. Indeed, multiple attempts at solubilizing DIP-α from tissue failed. Additionally, these experiments did not include cross-linking reagents, which could contribute to loss of weaker interactions such as Dpr-DIP binding. Overall, Nocte and cDIP are the first non-IgSF proteins to interact with the Dprs and DIPs in vivo and may be components of the Dpr10 signaling pathway.

### Nocte is required for motor neuron pathfinding

Nocte has been extensively studied in the adult fly for its role in circadian clock entrainment [14, 21, 50]; however, to our knowledge, no published studies have examined its function in other systems [20]. Our biochemical data in larval tissues suggest that Nocte may function in Dpr10-dependent processes. To examine Nocte function, we used *nocte*^*P*^, a hypomorphic allele generated by imprecise p-element excision [50].

**Fig. 3:**
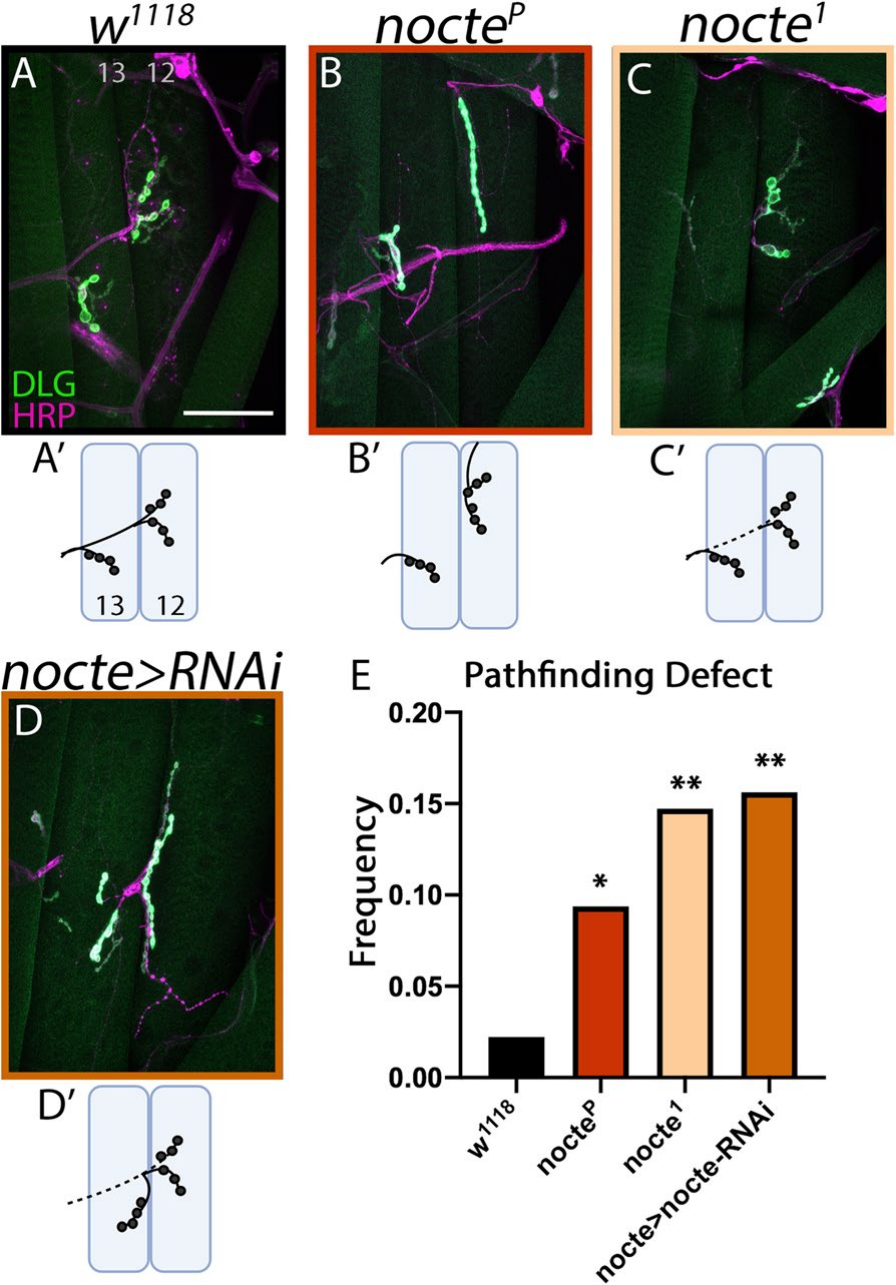
*nocte* mutants exhibit ISNb pathfinding errors. (**A-D**) m13 and m12 NMJs from respective genotypes. Neurons were visualized by staining for HRP (magenta) and the postsynapses were visualized by staining for DLG (green). Scale bar = 50 μm. (**A**,** A′**) Control *w*^*1118*^ animals with normal pathfinding (*n* = 90). Note that the ISNb travels above m13. (**B**,** B′**) *nocte*^*P*^ animals (red) revealed pathfinding defects (*n* = 192). Here, MN12-Ib traveled in a different nerve (likely the TN) and m12 lacked innervation by vCE. (**C**,** C′**) *nocte*^*1*^ animals (cream) showed innervation defects (*n* = 102). Here, m12 was innervated from below m13 and m13 is innervated by only the vCE not the Ib NMJ. (**D**,** D′**) *nocte-GAL4 x UAS-nocte-RNAix2* animals (cinnamon) also showed significant pathfinding defects (*n* = 96). Here, m12 and m13 are innervated after the nerve incorrectly traveled underneath m13. (**E**) Quantification of the frequency of pathfinding defects in the respective genotypes. Knockdown of *nocte* increased the frequency of ISNb pathfinding defects. *p < 0.05, **p < 0.01

In the neuromuscular junction, Dpr10 is required for m4 innervation by the dCE motor neuron [5] and ISNb pathfinding (Fig. 1). Based on our biochemical data (Fig. 2), Dpr10 may mediate these processes through interaction with Nocte. First, we compared m4 innervation in control and *nocte*^*P*^ mutants. In control larvae, the m4 innervation by the dCE occurred in 95.18% of hemisegments (*n* = 83) and *nocte*^*P*^ showed a similar penetrance (m4 innervation by the dCE occurred in 88.57% of hemisegments, *n* = 70, *p* = 0.145), suggesting that Nocte is not required for motor neuron-muscle synaptic partnerships at m4 (Supplementary Fig. 2A). We then examined ISNb pathfinding in control and *nocte*^*P*^ larvae. As discussed above, the trajectory and innervation of the ISNb is nearly invariant in control flies (Fig. 3A, E); however, in *nocte*^*P*^ hypomorphs this pattern is disrupted four-fold relative to controls (9.4% defect, *n* = 192, *p* = 0.043) (Fig. 3B, E). To confirm a role for Nocte in ISNb pathfinding, we examined a second allele, *nocte*^*1*^, a mutation that induces a stop codon after amino acid 1706 [50]. In this mutant background we observed a six-fold increase in defects relative to controls (14.7% of hemisegments, *n* = 102, *p* < 0.01). Similar to *nocte*^*P*^, *nocte*^*1*^ animals displayed normal m4 innervation by the dCE (91.30% of hemisegments, *n* = 69, *p* = 0.513) (Supplementary Fig. 2A). Finally, a previous study co-expressed two *nocte* RNAi constructs and showed a significant reduction in *nocte* RNA [50]. We used the same line to knockdown *nocte* using *nocte-GAL4* and found a seven-fold increase in ISNb misrouting (15.6%, *n* = 96, *p* < 0.01) of hemisegments examined (Fig. 3D-E). Similar to the hypomorphs, we observed ISNb pathfinding errors at m12 and m13 (Fig. 3A-D). Strikingly, disrupting *nocte* also resulted in muscle patterning defects similar to loss of *dpr10* (Supplementary Fig. 2B-E). Overall, these data reveal a novel role for Nocte in motor axon pathfinding, similar to those found in the putative upstream partner, Dpr10.

### *nocte* mutant embryos exhibit delayed nerve innervation

Motor axon pathfinding occurs during embryonic development when axons exit the VNC, navigate the periphery, and finally innervate their target muscles. The ISNb pathfinding defects we observed in *nocte* and *dpr10* mutants in third instar larvae could originate during the initial routing of axons or from axon retraction and subsequent misrouting in the larvae. To determine if pathfinding defects were present before larval development, we examined late stage 16 embryos, when most motor axon terminals have reached their target muscles.

**Fig. 4:**
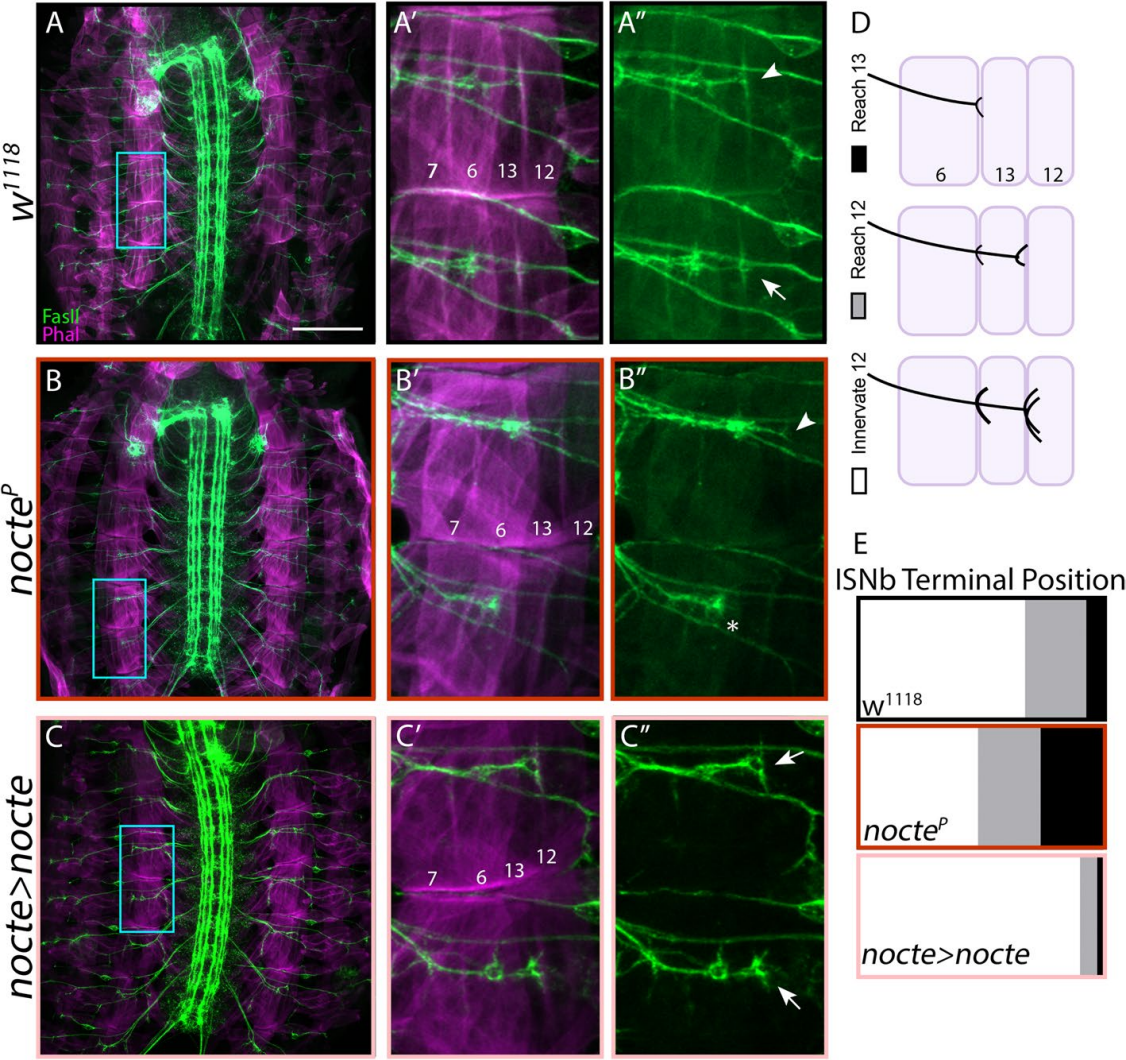
Loss of *nocte* causes stalling of ISNb before reaching terminal muscle targets. (**A-C**) Stage 16 embryo fillets stained for FasII (labels nerves, green) and phalloidin (labels muscles, magenta). Scale bar = 100 μm. (**A′-C′**) Insets from **A-C** (cyan rectangle), expanded to show ISNb terminal extension. (**A′-C″**) FasII stain of previous insets. Asterisk indicates terminal that reached and stalled at m13, arrowhead indicates terminal that traversed m13 and reached m12, and arrow indicates terminal that innervated m12. (**D**) Schematic of ISNb nerve terminals reaching or innervating m13 and m12. (**E**) Quantification of ISNb terminal position of genotypes in **A-C**. The bar graphs are represented as parts of a whole with the key given in **D**. Note that in *nocte*^*p*^ embryos the ISNb stalls at m13 with greater frequency than in either control or overexpression contexts (black bar)

Indeed, *nocte*^*P*^ embryos exhibited ISNb pathfinding defects. To delineate ISNb development more accurately in control and mutant larvae, we used immunohistochemistry against Fasciclin 2 to determine the ISNb terminal position and scored the ISNb as i) reaching m13, ii) traversing m13 and reaching m12, and iii) innervating m12. An axon terminal was classified as reaching its target when filipodia extended along the muscle edge, and innervation occurred once varicosity-like structures appeared over the muscle surface [62], (Fig. 4D). In late stage 16 control embryos, 6.82% of ISNb terminals reached m13, 25% traversed m13 and reached m12, and 68.18% formed varicosity-like structures on m12 (Fig. 4A, E; *n* = 44) compared to 25.53, 25.53, and 48.93%, respectively, in *nocte*^*P*^ embryos. Thus, perturbing *nocte* resulted in ISNb pausing, or stalling, and delayed m12 innervation (Fig. 4B, E; *n* = 47, *p* < 0.0001). On the other hand, overexpression of *nocte* expedited ISNb pathfinding and innervation; 2.33% of ISNb terminals reached m13, 6.98% traversed m13 and reached m12, and 90.70% innervated m12 (Fig. 4C, E; *n* = 43, *p* < 0.01). Taken together, these data suggest that Nocte is required for axonal pathfinding at embryonic stages and that *nocte* mutant growth cones reach their targets more slowly than controls.

### *nocte* is expressed in subsets neurons, muscles, and glia

Motor axon pathfinding requires interactions between neurons, muscles, and glia [2, 26, 58]. To understand how Nocte controls ISNb axon pathfinding, we examined the expression pattern of *nocte*.

**Fig. 5:**
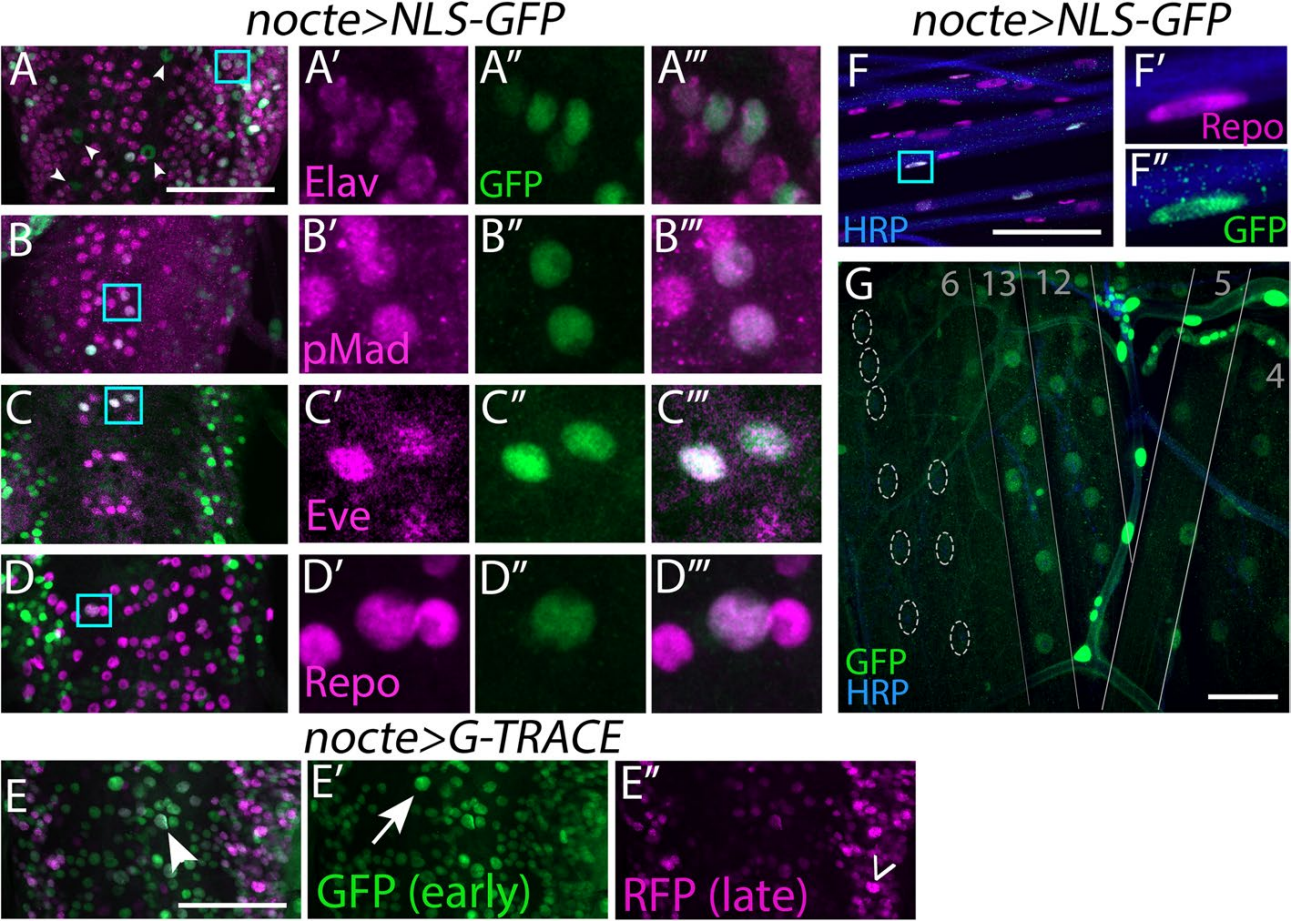
*nocte* is broadly expressed in larvae. (**A-D**) Larval VNCs from *nocte-GAL4 x UAS-NLS-GFP* animals stained for GFP (green) and cell specific markers (magenta). (**A′-D‴**) Insets of **A-D** (cyan rectangles) showing co-localization of GFP and the cell specific markers. (**E-E″**) VNC of *nocte-GAL4 x UAS-G-TRACE* larva stained for GFP (green) indicating only early expression (arrow) and RFP (magenta) indicating only late expression (caret). Cell expressing both GFP and RFP (arrowhead) suggest *nocte* is constitutively expressed. (**F-G**) *nocte-GAL4 x UAS-NLS-GFP* animals stained for GFP (green) and neuronal tissue (HRP, blue). (**F-F″**) Peripheral nerves stained for the glial marker Repo (magenta). (**F′-F″**) Insets of cyan rectangle in **F**. Note that only a subset of glial cells express *nocte*. (**G**) Ventral muscle field showing expression in m13, 12, 5, 4 but not m6 (nuclei lacking GFP outlined in dotted ellipses). All scale bars = 50 μm

To examine *nocte* expression in larvae, we used *nocte-GAL4* [50] to drive a nuclear localized green fluorescent protein (*nocte > NLS-GFP*), and cell types were verified by co-staining with specific markers. *Nocte* was widely expressed in the larval VNC (Fig. 5A-E). Co-staining with Elav, a panneuronal marker, revealed substantial overlap with GFP, suggesting that *nocte* is expressed in neurons (Fig. 5A). Moreover, co-staining with a pan-motor neuron marker pMad and the transcription factor Eve revealed that a subset of motor neurons, including dCE and aCC, and interneurons express *nocte*. (Fig. 5B-C). Finally, some *nocte* + cells in the VNC were not labeled with Elav, suggesting that *nocte* is also expressed in non-neuronal cells (Fig. 5A, arrowheads); co-staining with the glial maker, Repo, identified these as glial cells (Fig. 5D).

The analyses above only revealed *nocte* expression in 3rd instar larvae. However, Nocte is required for pathfinding, suggesting that it is expressed in earlier developmental stages. To examine *nocte* temporal expression, we used the G-TRACE system to permanently label a cell with one fluorophore (GFP) and transiently with another fluorophore (RFP) [18, 56]. Thus, cells that are only GFP+ expressed *nocte* earlier in development. In the larval VNC, we observed broad overlap between red and green nuclei but also GFP+ only nuclei, suggesting that nocte is temporally expressed in earlier developmental stages (Fig. 5E).

We also characterized *nocte* expression outside the VNC. In *nocte > NLS-GFP* larvae, we observed GFP+ nuclei along peripheral nerves where glial cell bodies reside, and we confirmed glial expression by co-localization with Repo (Fig. 5F). However, only a subset of glial cells expressed *nocte*. Examination of the larval body wall revealed *nocte* expression in a large subset of muscles (Fig. 5G), including m13 and m12. Furthermore, we found a cluster of GFP+ cells posterior to m8 (Supplementary Fig. 3A). Based on the location of these cells, the absence of Repo staining, and overlap with Cut staining, we identified these cells as adult muscle precursor cells (AMPs), specifically Lateral-AMPs [19, 33]. Finally, *nocte* is expressed in other tissues including the fat body, and gut (Supplementary Fig. 3B-C). Similar to peripheral glia, only a subset of fat body cells expressed *nocte* (Supplementary Fig. 3B), suggesting that *nocte* can subdivide fat body tissue at the molecular level. However, in the embryonic VNC, *nocte* is more ubiquitously expressed (Supplementary Fig. 3D).

Taken together, *nocte* is expressed in subsets of cells in a variety of larval tissues including the nervous system, muscles, and the fat body with subset of these cells temporally expressing *nocte* throughout development.

### Nocte localizes to the cytoplasm and nucleus

**Fig. 6:**
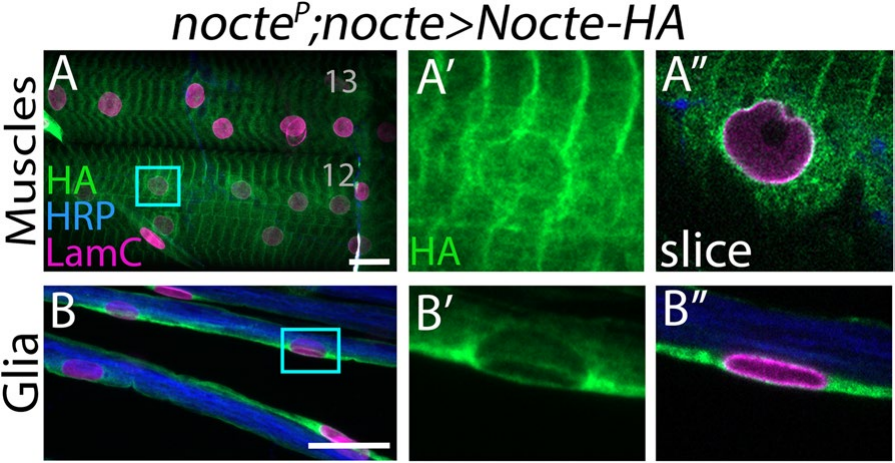
Sub-cellular localization of Nocte. (**A, B**) *UAS-Nocte-HA* was expressed by *nocte-GAL4* in a *nocte*^*P*^ mutant background. Nuclear lamina was stained with LamC (magenta), neuronal tissue was visualized by staining with HRP (blue), and Nocte was localized by staining for HA (green). (**A**) In m12 and m13, Nocte was localized around the nucleus and in striated bands. (**B**) In nerves, Nocte was localized in glial cells surrounding axons. (**A′-B′**) Inset of **A-B** (cyan rectangle), enlarging a nucleus and showing only the green channel. (**A″-B″**) Single z-plane confocal slice of nucleus in **A′-B′** with all channels present. All scale bars = 20 μm

We lack insight into where Nocte is found inside the cell. Structural prediction of Nocte using Alphafold did not reveal any protein domains [28] (Supplementary Fig. 4). Nocte lacks a signal peptide, suggesting that it functions intracellularly. To gain insight into Nocte sub-cellular localization, we used a *UAS-HA-nocte* transgenic line [13]. Expression of *HA-nocte* with *nocte-GAL4* resulted in lethality at larval and pupal stages; to circumvent this, we overexpressed *HA-nocte* in a *nocte*^*P*^ hypomorphic background. We used antibodies against HA to visualize Nocte localization and found significant staining around the nuclear envelope marker, LamC, in both glia and muscle cells (Fig. 6A-B). In addition, we observed that Nocte localizes in striated patterns in the muscle (Fig. 6A), mirroring the alternating muscle A- and I-bands [46]. Thus, these experiments suggest that Nocte is distributed throughout the cytoplasm and is concentrated around the nucleus.

### Nocte is required in neurons to instruct axon pathfinding

**Fig. 7:**
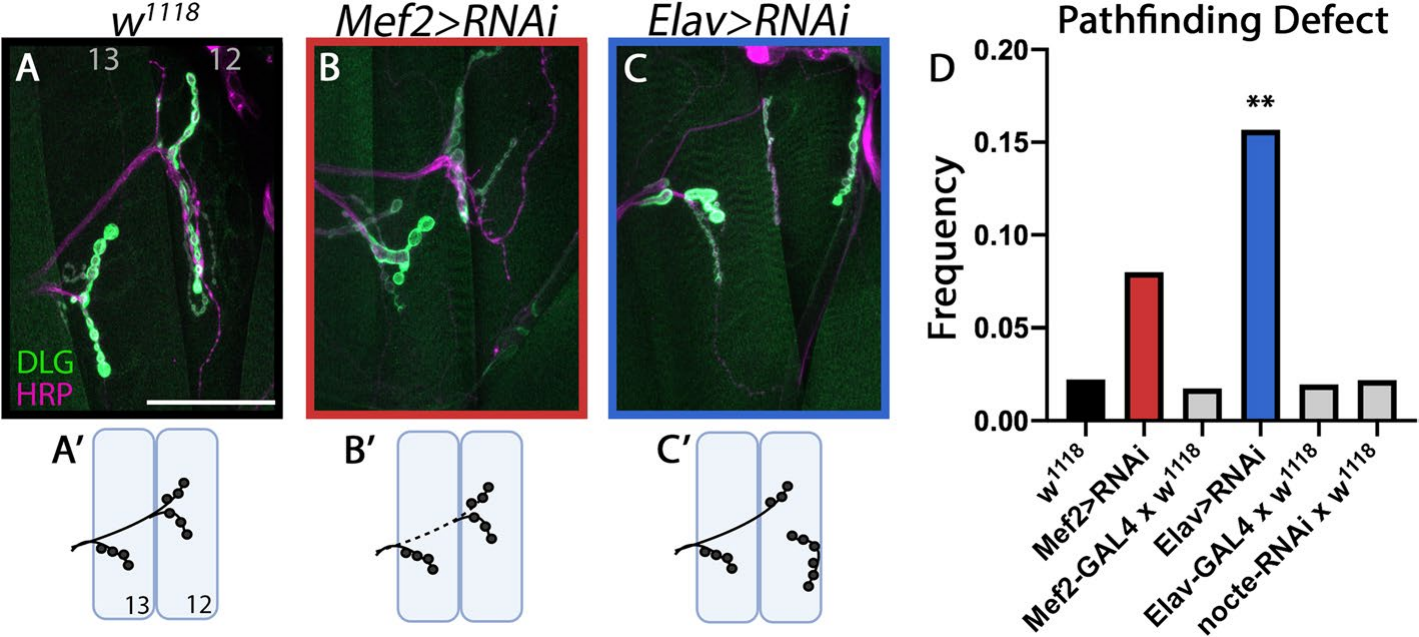
Tissue specific knockdown of *nocte* using RNAi. (**A-A′**) *w*^*1118*^ control animals depicting the normal ISNb projection over m13 before innervating m12 (*n* = 90). Neurons were labeled by staining for HRP (magenta) and postsynapses labeled by staining for DLG (green). Scale bar = 50 μm. (**B-B′**) *Mef2-GAL4 x UAS-nocte-RNAix2* results in modest misrouting (*n* = 125). Here, the m12 ISNb branch for travels under m13. (**C-C′**) *Elav-GAL4 x UAS-nocte-RNAix2* led to significant misrouting of the ISNb (*n* = 134). Here, the MN12-Ib innervates from the dorsal side rather than the ventral side. (**D**) Quantification of the frequency of ISNb pathfinding defects in the respective genotypes. Note that knockdown of *nocte* in neurons (*Elav > RNAi*) significantly increased pathfinding defects. **p < 0.01

To determine where Nocte functions for motor axon pathfinding, we used RNAi to knock down *nocte* pre- and postsynaptically. Reducing *nocte* levels in muscles showed an increasing trend of ISNb pathfinding compared to controls but they were not statistically significant (*Mef2 > nocte-RNAi* = 8.0%, *n* = 125, *p* = 0.079; Fig. 7A-B, D). However, many of these animals displayed muscle patterning defects with concomitant innervation defects (Supplementary Fig. 5). Knockdown of *nocte* in neurons lead to a significant seven-fold increase in ISNb pathfinding defects (*w*^*1118*^ = 2.2%, *n* = 135; *Elav > nocte-RNAi* = 15.7%, *n* = 134, *p* < 0.001) (Fig. 7A, C, D). Knockdown of *nocte* in all *nocte* expressing cells (*nocte-GAL4*) resulted in a similar seven-fold increase in ISNb pathfinding errors (15.6%, *n* = 96, *p* < 0.001) compared to neuronal knockdown (Fig. 3D-E). Neither *GAL4* nor the *UAS-nocte-RNAi* alone had significant pathfinding defects (Fig. 7D). Overall, these data suggest that Nocte functions presynaptically in motor neurons to mediate ISNb pathfinding, and postsynaptic Nocte may regulate muscle patterning.

### Nocte and Dpr10 genetically interact

Dpr10 is a cell surface protein, and like Nocte, it is expressed in motor neurons and muscles [5, 56]. Dpr10 and Nocte also interact biochemically in vivo (Fig. 2) and loss of each resulted in ISNb pathfinding defects. Here, we tested whether Dpr10 and Nocte function in the same pathway.

**Fig. 8:**
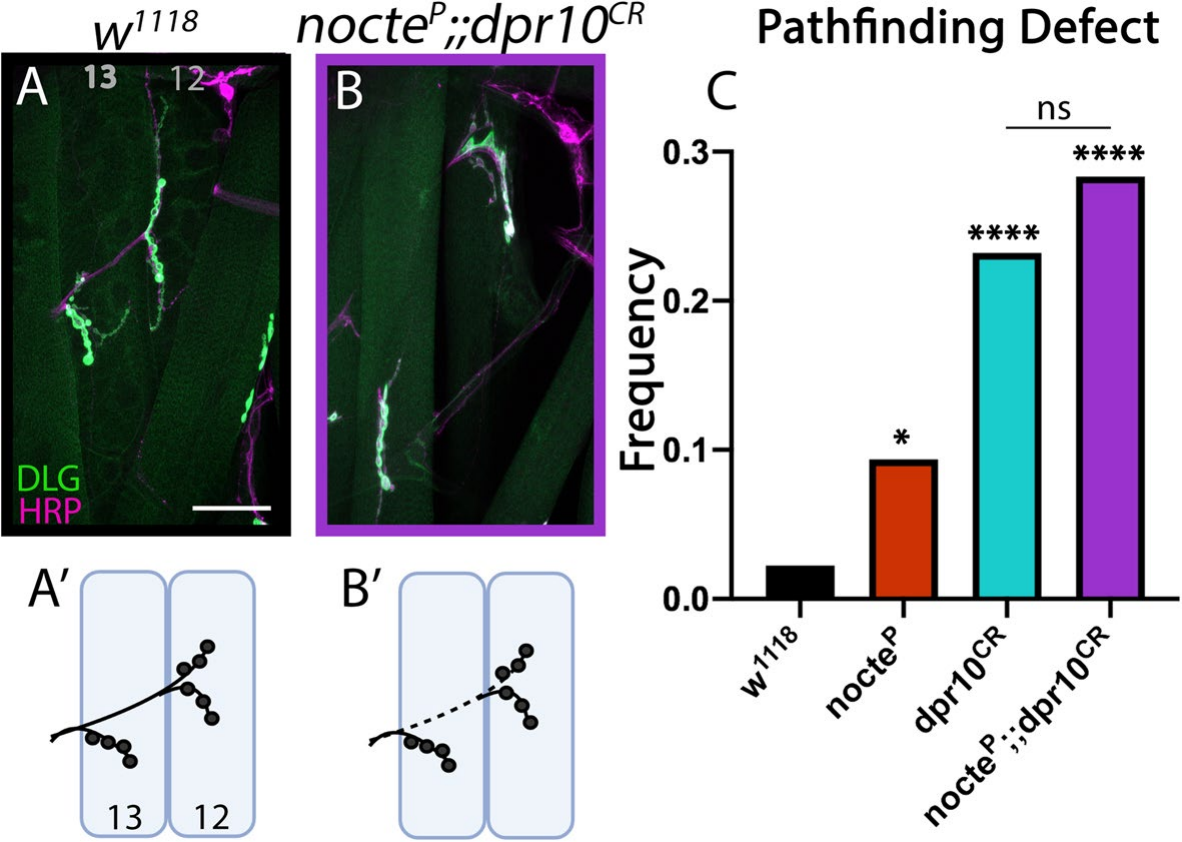
*nocte*^*P*^ and *dpr10*^*CR*^ double mutants are indistinguishable from *dpr10*^*CR*^ single mutants. NMJs of m13 and m12, with neurons shown in magenta (HRP) and postsynapses in green (DLG). Scale bar = 50 μm. (**A-A′**) Normal pathfinding in a *w*^*1118*^ control animal (*n* = 90). (**B-B′**) In a *nocte,dpr10* double mutant, the ISNb traveled under m13 before innervating m12 (*n* = 120). (**C**) Quantification of misrouting frequencies of control, single, and double mutant animals. *p < 0.05, ****p < 0.0001

*dpr10* mutants alone exhibited ISNb pathfinding and innervation defects at levels higher than *nocte* mutants (Fig. 8). Specifically, *dpr10*^*CR*^ larvae displayed innervation defects in 23.2% of examined hemisegments (*n* = 90) compared to 9.4% in *nocte*^*P*^ larvae (*n* = 192) (Fig. 8C). We reasoned that if *dpr10* and *nocte* are in the same pathway, double mutants would exhibit defects similar to the single *dpr10* mutant. Indeed, in *nocte*^*P*^*;;dpr10*^*CR*^ double mutants, ISNb misrouting was not statistically different than *dpr10*^*CR*^ mutants (28.3%, *n* = 120, *p* = 0.584) (Fig. 8B-C). Moreover, the double mutants exhibited muscle patterning defects akin to single mutant animals (Supplementary Fig. 6). Taken together, these data suggest that Dpr10 and Nocte function in the same pathway to instruct ISNb pathfinding and innervation.

## Discussion

The Dpr and DIP subfamilies of the IgSF have been implicated in several steps of *Drosophila* nervous system development. In this study, we revealed a novel function for Dpr10 in motor axon pathfinding. To understand the Dpr10 molecular pathway that underlies this role, we identified Nocte as a novel Dpr10 interactor. In support of Dpr10 and Nocte being in the same molecular pathway, loss of *nocte* led to ISNb pathfinding defects, and *nocte,dpr10* double mutants did not exacerbate the *dpr10* phenotype. Knockdown of *nocte* in neurons caused significant axon misrouting, suggesting that Nocte and Dpr10 interact presynaptically to mediate ISNb pathfinding. Overall, our data identified new interactors of Dpr10 and revealed novel roles for Nocte and Dpr10 in motor neuron pathfinding.

### Examining Dpr-DIP mediated mechanisms

Several CSP families have been implicated in circuit assembly, but the signaling pathways are much less understood. The *Drosophila* Dprs and DIPs instruct synaptic connectivity in the optic lobe and neuromuscular junction and other neurodevelopmental processes including motor axon terminal morphology, olfactory axon pathfinding, and cell survival. In this study, we demonstrate that Dpr10 is also required for motor axon pathfinding. Thus, in the neuromuscular system, Dpr10 is used for motor axon pathfinding (this study) and later for synaptic connectivity [5]. Together, these studies suggest that Dprs and DIPs may use multiple signaling pathways to mediate their pleotropic functions.

At the larval NMJ, loss of *dpr11* and *DIP-γ* revealed exuberant satellite boutons [10], reminiscent of BMP signaling overactivation [41, 42]. Genetic interaction tests and BMP pathway reporters confirmed that *dpr11* and *DIP-γ* modulate BMP signaling [10]. How Dprs and DIPs interact with BMP ligands and/or receptors in not known. The vertebrate orthologs of the Dprs and DIPs, the IgLONs, are required for normal neurite outgrowth in forebrain [3] and hippocampal neurons [53]. One member of the IgLON family, Negr1, signals through the FGF pathway to promote neuronal arborization [45]. In the *Drosophila* larval neuromuscular junction, one of the FGF receptors, Heartless, is required for NMJ development [51]. Whether the Dpr/DIP and FGF pathways interact is not known. Elucidating signaling pathways of the Dprs and DIPs may contribute to our understanding of IgLON mechanisms during vertebrate nervous system development.

To uncover potential Dpr/DIP signaling pathways, we sought to identify proteins that interact with Dpr10. We immunoprecipitated Dpr10 and found several interactors including cDIP, an LRR protein previously identified in an in vitro screen as a Dpr10 binding protein [10, 43]. cDIP binds many Dprs and DIPs, and in the *Drosophila* visual system, cDIP is required in glial cells for synaptic refinement [52]; a role in the neuromuscular junction has not been reported. cDIP may be involved in a Dpr10-mediated pathway, but as a putative secreted protein, it would not be able to signal directly into the cell.

Another Dpr10 interactor identified in our screen was Nocte, a glutamine-rich protein that is predicted to be an unstructured intracellular protein. While overexpression of a protein can lead to off-target interactions, Nocte was found to interact with Dpr10 in two different tissues, suggesting the interaction is not merely due to excessive Dpr10 expression. Previous work implicated Nocte in temperature-dependent entrainment of circadian rhythm [50]. In this study, we collected body wall and brain tissues to perform Dpr10 immunoprecipitations, and in both samples, we isolated Nocte; however, we cannot distinguish between direct or indirect interactions. The association between Dpr10 and Nocte, whether direct or indirect, suggests that Nocte must also localize at or near the cell membrane. A previous study found that in the adult fly head, Nocte was enriched in the membrane associated fraction of a mass spectrometry experiment [4], suggesting that Nocte endogenously binds proteins at or in the cell membrane. Furthermore, when Nocte was expressed with *timeless-GAL4* in adult fly heads and immunoprecipitated, the transmembrane receptor Ir25a was identified as an interactor. Thus, Nocte is known to interact with membrane associated proteins.

Functionally, *dpr10* and *nocte* mutants exhibited similar pathfinding defects and *nocte,dpr10* double mutants displayed misrouting at a similar frequency to *dpr10* single mutants, suggesting these two genes are in the same ISNb pathfinding pathway. How they work together at a mechanistic level is unknown but binding of Dpr10 with an extracellular ligand could modify its interaction with Nocte to activate or inhibit downstream signaling.

### ISNb pathfinding is mediated by multiple pathways

During axon pathfinding, growth cones must recognize multiple attractive and repulsive cues that instruct the axons to their appropriate destinations. In addition to the challenges of interpreting these cues, some axons must traverse long distances to find their correct targets [26, 30]. The synaptic connections that are finally established are critical for all behaviors, learning, and memory, and thus, axon pathfinding is tightly regulated. Identifying the cues that regulate this process is challenging, in part, because of molecular redundancy [54]. In *Drosophila* embryos, the ISNb innervates muscles 7, 6, 28, 30, 14, 13, and 12. However, the cell bodies for the axons in the ISNb are located in the VNC. These axons must exit the VNC in defined locations, ignore incorrect target cells, make precise decisions about where to defasciculate, and ultimately innervate their respective muscles. Disruption of axon pathfinding can cause defects in any of these steps. Previous studies have implicated multiple signaling pathways in ISNb pathfinding, and not surprisingly, many of the identified genes code for CSPs.

In [32], the Zinn lab conducted a screen of CSPs that mediate synaptic pathfinding and target selection. Loss- and gain-of-function analyses revealed multiple LRR proteins, such as Tartan (Trn), Capricious (Caps), and Hattifattener (Haf), as mediators of ISNb pathfinding. For example, manipulation of *trn* levels showed multiple ISNb mistargeting and pathfinding phenotypes in embryos and larvae including stalling and misrouting under m13 and m12 leading to innervation of m12 on the dorsal side. These defects mimic those we observed after loss of *dpr10* and *nocte*. Moreover, the same study reported similar frequencies of the misrouting defect, with *caps* mutants exhibiting ~ 15% defects, and *trn* nulls mistargeting in ~ 40% of hemisegments. Interestingly, loss of *trn* and *caps* also lead to muscle patterning defects, akin to loss of *dpr10* and *nocte*.

In another study, Tolloid-related 1 (Tlr1) was also implicated in motor axon pathfinding. In *tlr1* mutants, m12 is incorrectly innervated from the dorsal rather than the ventral side [39]. Tlr1 is a metalloprotease that functions together with Sidestep, an IgSF CSP, for proper ISNb defasciculation [39]. Taken together, these studies and our work support the model that multiple CSP families act combinatorically and/or redundantly to mediate ISNb pathfinding.

### Putative function of Nocte in axon pathfinding

Our current study identified Nocte as an interactor of Dpr10 in larval brains and body walls. *Nocte* is widely expressed in the larva and distributed throughout the cytoplasm based on localization of a transgenic Nocte-HA; however, overexpression and the HA tag could mislocalize Nocte so caution must be taken in interpreting these results. To gain insight into Nocte structure and function, we used the structure prediction software AlphaFold [28]; however, it was unable to resolve clear domains for Nocte. Other prediction software programs (Rosetta, RaptorX) were unable to run predictions as Nocte is a relatively large protein with 2309 amino acids. Given the lack of clear structure, we cannot infer the molecular function of Nocte or how it may bind Dpr10.

Based on our genetic and biochemical data, Nocte functions in either a repulsive or attractive axon pathfinding cascade mediated through Dpr10. In either model, when Nocte is removed the nerve cannot distinguish between the correct and incorrect trajectories. Similar observations were made when disrupting the repulsive Semaphorin-Plexin pathway: loss of either the ligand or receptor perturbs axon defasciculation and axon guidance [31, 57]. Similarly, when the attractive cue Netrin, or its receptor Unc5, is removed, defasciculation does not occur and the motor neurons that reach the periphery fail to innervate their target muscles properly [29, 40].

In vertebrates, Proline rich coiled-coil 2a (Prrc2a) is the ortholog of Nocte [25] and functions as a N^6^-methyladenosine (m^6^A) reader [59]. m^6^A is the most abundant mRNA modification found in eukaryotes and regulates mRNA stability, splicing, and translation [27]. In glial cells, Prrc2a modulates mRNA stability and is required for cell specification and maintenance [59]. In *Drosophila*, m^6^A modifications are critical for sex determination and neuronal function and are upregulated during embryogenesis and pupal stages [23, 36]. If Nocte is an m^6^A reader, Nocte could regulate the temporal expression of pathfinding molecules at specific choice points. In support of this model, *nocte* knockdown and overexpression delayed and expedited, respectively, the innervation of m12 by the ISNb. In a similar model, the Hox gene *Ultrabithorax* (*Ubx*) regulates ISNb routing by controlling expression of pathfinding molecules [24].

An open question is why Nocte interacts with cell surface CSPs, including Dpr10 and Ir25a. One possibility is that interactions with CSPs may regulate Nocte by sequestering it. Alternatively, CSPs could transduce an extracellular signal to Nocte to regulate relevant mRNAs. Future studies will examine if Nocte, like its vertebrate ortholog Prrc2a, regulates mRNAs though m^6^A modifications. Moreover, it is unknown how Nocte associates with Ir25a and Dpr10, and if these interactions influence Nocte function. Taken together, this study identified in vivo interactors of Dpr10 and novel functions for Nocte and Dpr10 in motor axon pathfinding.

## Supplementary Information

**Additional file 1: Supplementary Fig. 1** (Supplement to Fig. 1): Embryo hatching and muscle patterning defects in *dpr10* mutants. (**A**) Percentage of hatched embryos in *w*^*1118*^ and *dpr10*^*CR*^ backgrounds, ***p < 0.001. (**B**) A control *w*^*1118*^ animal depicting the normal muscle patterns in the ventral field. Neurons were labeled by staining for HRP (magenta) and postsynapses were labeled by staining for DLG (green). Note that the GFP channel also outlines individual muscles. (**C**) A *dpr10*^*CR*^ animal with a missing m5. Ectopic innervation of m12 by MN5-Ib indicated with arrowhead. (**D**) A *dpr10-GAL4* animal with a split m5. (**B′**-**D′**) Cartoon schematics of muscle patterning observed in **A**-**C**. Aberrant muscles highlighted in red. Scale bar = 50 μm.

**Additional file 2: Supplementary Fig. 2** (Supplement to Fig. 3): *nocte* mutants exhibit normal innervation of m4 but display muscle defects. (**A**) Innervation frequency of m4 by the dCE (Is neuron that innervates the dorsal muscles) in respective genotypes. Loss of *nocte* does not affect dCE innervation of m4. ****p < 0.0001. (**B**) A control *w*^*1118*^ animal with normal muscle patterns. Neurons were labeled by staining for HRP (magenta) and postsynapses were labeled by staining for DLG (green). (**C**) A *nocte*^*1*^ animal with a split m5. (**D**) A *nocte*^*P*^ animal with duplicated m13 or m12. (**E**) A *nocte-GAL4 x nocte-RNAi* animal with a triplicated m5. (**B′**-**E′**) Cartoon schematics of muscle patterns observed in **B**-**E**. Aberrant muscles shown in red. Scale bar = 50 μm.

**Additional file 3: Supplementary Fig. 3** (Supplement to Fig. 5): Nocte peripheral expression. (**A**) *nocte-GAL4 x UAS-NLS-GFP* larvae. Lateral adult muscle precursor cells (L-AMPs) highlighted by a yellow rectangle. Preps were co-stained for the transcription factor Cut (magenta) and HRP (blue). (**A′**) GFP channel. (**B**-**D**) *nocte-GAL4 x UAS-nocte-HA* larvae. (**B**) Larval fat body cells. Nocte localization labeled with HA (green) and FB outline shown in HRP (blue). Cells lacking *nocte* expression indicated with arrowhead. (**C**) Embryonic gut dissected away from rest of embryo. Nocte localization labeled with HA (green) and gut outline shown in HRP (blue). (**D**) In the embryonic VNC, transgenic Nocte expression visualized with HA (green), nerves labeled with FasII (magenta), and muscles labeled with phalloidin (blue). All scale bars = 50 μm.

**Additional file 4: Supplementary Fig. 4** (Supplement to Fig. 6): AlphaFold structural prediction of Nocte. The orange lines are predicted unstructured regions with fragments of alpha helices visible in the center.

**Additional file 5: Supplementary Fig. 5** (Supplement to Fig. 7): Muscle patterning defects caused by knockdown of *nocte* in muscles. (**A**) A control *w*^*1118*^ animal depicting the normal muscle pattern. Neurons are labeled by HRP staining (magenta) and postsynapses by DLG staining (green). Note that the outline of muscles can be clearly visualized in the green channel. Scale bar = 50 μm. (**B**-**D**) *Mef2-GAL4 x UAS-nocte-RNAix2* animals revealed several defects including (**B**) crisscrossing of m6 and m7, (**C**) duplication of m5, and (**D**) absence of m5. (**A′**-**D′**) Cartoon schematics of muscle patterns observed in **A**-**D**. Aberrant muscles shown in red. Scale bar = 50 μm.

**Additional file 6: Supplementary Fig. 6** (Supplement to Fig. 8): Muscle defects in *nocte,dpr10* double mutants. (**A**) A control *w*^*1118*^ animal depicting the normal muscle pattern. Neurons are labeled by HRP staining (magenta) and postsynapses by DLG staining (green). Note that the outline of muscles can be clearly visualized in the green channel. Scale bar = 50 μm. (**B**) *nocte*^*P*^*;;dpr10*^*CR*^ double mutant animals showed various muscle patterning defects including duplication of m13 or m12 and missing m5. (**A′**-**B′**) Cartoon schematics of muscle patterns observed in **A**-**B**. Aberrant muscles shown in red. Scale bar = 50 μm.

## Data Availability

The datasets generated during and analyzed during the current study are available from the corresponding author.

## References

[1] Aberle H (2019). Axon guidance and collective cell migration by substrate-derived attractants. Front Mol Neurosci.

[2] Abrell S, Jäckle H (2001). Axon guidance of Drosophila SNb motoneurons depends on the cooperative action of muscular Krüppel and neuronal capricious activities. Mech Dev.

[3] Akeel M, McNamee CJ, Youssef S, Moss D (2011). DIgLONs inhibit initiation of neurite outgrowth from forebrain neurons via an IgLON-containing receptor complex. Brain Res.

[4] Aradska J, Bulat T, Sialana FJ, Birner-Gruenberger R, Erich B, Lubec G (2015). Gel-free mass spectrometry analysis of Drosophila melanogaster heads. Proteomics.

[5] Ashley J, Sorrentino V, Lobb-Rabe M, Nagarkar-Jaiswal S, Tan L, Xu S, Xiao Q, Zinn K, Carrillo RA (2019). Transsynaptic interactions between IgSF proteins DIP-α and Dpr10 are required for motor neuron targeting specificity. Elife.

[6] Barish S, Nuss S, Strunilin I, Bao S, Mukherjee S, Jones CD, Volkan PC (2018). Combinations of DIPs and Dprs control organization of olfactory receptor neuron terminals in Drosophila. PLoS Genet.

[7] Blockus H, Chédotal A (2016). Slit-Robo signaling. Development.

[8] Bornstein B, Meltzer H, Adler R, Alyagor I, Berkun V, Cummings G, Reh F, Keren-Shaul H, David E, Riemensperger T, Schuldiner O (2021). Transneuronal Dpr12/DIP-δ interactions facilitate compartmentalized dopaminergic innervation of Drosophila mushroom body axons. EMBO J.

[9] Boyer NP, Gupton SL (2018). Revisiting Netrin-1: one who guides (axons). Front Cell Neurosci.

[10] Carrillo RA, Özkan E, Menon KP, Nagarkar-Jaiswal S, Lee PT, Jeon M, Birnbaum ME, Bellen HJ, Garcia KC, Zinn K (2015). Control of synaptic connectivity by a network of Drosophila IgSF cell surface proteins. Cell.

[11] Cash S, Chiba A, Keshishian H (1992). Alternate neuromuscular target selection following the loss of single muscle fibers in Drosophila. J Neurosci.

[12] Chang TN, Keshishian H (1996). Laser ablation of Drosophila embryonic Motoneurons causes ectopic innervation of target muscle fibers. J Neurosci.

[13] Chen C, Buhl E, Xu M, Croset V, Rees JS, Lilley KS, Benton R, Hodge JJL, Stanewsky R (2015). Drosophila ionotropic receptor 25a mediates circadian clock resetting by temperature. Nature.

[14] Chen C, Xu M, Anantaprakorn Y, Rosing M, Stanewsky R (2018). Nocte is required for integrating light and temperature inputs in circadian clock neurons of Drosophila. Curr Biol.

[15] Cosmanescu F, Katsamba PS, Sergeeva AP, Ahlsen G, Patel SD, Brewer JJ, Tan L, Xu S, Xiao Q, Nagarkar-Jaiswal S, Nern A, Bellen HJ, Zipursky SL, Honig B, Shapiro L (2018). Neuron-subtype-specific expression, interaction affinities, and specificity determinants of DIP/Dpr cell recognition proteins. Neuron.

[16] Courgeon M, Desplan C (2019). Coordination between stochastic and deterministic specification in the Drosophila visual system. Science.

[17] Dickson BJ, Gilestro GF (2006). Regulation of commissural axon pathfinding by slit and its Robo receptors. Cell Dev Biol.

[18] Evans CJ, Olson JM, Ngo KT, Kim E, Lee NE, Kuoy E, Patananan AN, Sitz D, Tran P, Do M-T, Yackle K, Cespedes A, Hartenstein V, Call GB, Banerjee U (2009). G-TRACE: rapid Gal4-based cell lineage analysis in Drosophila. Nat Methods.

[19] Figeac N, Jagla T, Aradhya R, Ponte JPD, Jagla K (2010). Drosophila adult muscle precursors form a network of interconnected cells and are specified by the rhomboid-triggered EGF pathway. Development.

[20] George R, Stanewsky R (2021). Peripheral sensory organs contribute to temperature synchronization of the circadian clock in Drosophila melanogaster. Front Physiol.

[21] Glaser FT, Stanewsky R (2005). Temperature synchronization of the Drosophila circadian clock. Curr Biol.

[22] Guan B, Hartmann B, Kho Y-H, Gorczyca M, Budnik V (1996). The Drosophila tumor suppressor gene, dlg, is involved in structural plasticity at a glutamatergic synapse. Curr Biol.

[23] Haussmann IU, Bodi Z, Sanchez-Moran E, Mongan NP, Archer N, Fray RG, Soller M (2016). m6A potentiates Sxl alternative pre-mRNA splicing for robust Drosophila sex determination. Nature.

[24] Hessinger C, Technau GM, Rogulja-Ortmann A (2016). The Drosophila Hox gene Ultrabithorax acts in both muscles and motoneurons to orchestrate formation of specific neuromuscular connections. Development.

[25] Hu Y, Flockhart I, Vinayagam A, Bergwitz C, Berger B, Perrimon N, Mohr SE (2011). An integrative approach to ortholog prediction for disease-focused and other functional studies. BMC Bioinformatics.

[26] Jeong S (2021). Molecular mechanisms underlying motor axon guidance in Drosophila. Mol Cells.

[27] Jiang X, Liu B, Nie Z, Duan L, Xiong Q, Jin Z, Yang C, Chen Y (2021). The role of m6A modification in the biological functions and diseases. Signal Transduct Target Ther.

[28] Jumper J, Evans R, Pritzel A, Green T, Figurnov M, Ronneberger O, Tunyasuvunakool K, Bates R, Žídek A, Potapenko A, Bridgland A, Meyer C, Kohl SAA, Ballard AJ, Cowie A, Romera-Paredes B, Nikolov S, Jain R, Adler J, Back T, Petersen S, Reiman D, Clancy E, Zielinski M, Steinegger M, Pacholska M, Berghammer T, Bodenstein S, Silver D, Vinyals O, Senior AW, Kavukcuoglu K, Kohli P, Hassabis D (2021). Highly accurate protein structure prediction with AlphaFold. Nature.

[29] Keleman K, Dickson BJ (2001). Short- and long-range repulsion by the Drosophila Unc5 netrin receptor. Neuron.

[30] Kolodkin AL, Tessier-Lavigne M (2011). Mechanisms and molecules of neuronal wiring: a primer. Csh Perspect Biol.

[31] Koropouli E, Kolodkin AL (2014). Semaphorins and the dynamic regulation of synapse assembly, refinement, and function. Curr Opin Neurobiol.

[32] Kurusu M, Cording A, Taniguchi M, Menon K, Suzuki E, Zinn K (2008). A screen of cell-surface molecules identifies leucine-rich repeat proteins as key mediators of synaptic target selection. Neuron.

[33] Lavergne G, Zmojdzian M, Ponte JPD, Junion G, Jagla K (2020). Drosophila adult muscle precursor cells contribute to motor axon pathfinding and proper innervation of embryonic muscles. Development.

[34] Lee H-KP, Wright AP, Zinn K. Live dissection of Drosophila embryos: streamlined methods for screening mutant collections by antibody staining. J Vis Exp. 2009. 10.3791/1647. 10.3791/1647 PMC3149970 20040910

[35] Lee P-T, Zirin J, Kanca O, Lin W-W, Schulze KL, Li-Kroeger D, Tao R, Devereaux C, Hu Y, Chung V, Fang Y, He Y, Pan H, Ge M, Zuo Z, Housden BE, Mohr SE, Yamamoto S, Levis RW, Spradling AC, Perrimon N, Bellen HJ (2018). A gene-specific T2A-GAL4 library for Drosophila. Elife.

[36] Lence T, Akhtar J, Bayer M, Schmid K, Spindler L, Ho CH, Kreim N, Andrade-Navarro MA, Poeck B, Helm M, Roignant J-Y (2016). m6A modulates neuronal functions and sex determination in Drosophila. Nature.

[37] Maness PF, Schachner M (2007). Neural recognition molecules of the immunoglobulin superfamily: signaling transducers of axon guidance and neuronal migration. Nat Neurosci.

[38] Menon KP, Kulkarni V, Shin-Ya T, Anaya M, Zinn K (2019). Interactions between dpr11 and dip-y control election of amacrine neurons in drosophila color ision circuits. Elife.

[39] Meyer F, Aberle H (2006). At the next stop sign turn right: the metalloprotease Tolloid-related 1 controls defasciculation of motor axons in Drosophila. Development.

[40] Mitchell KJ, Doyle JL, Serafini T, Kennedy TE, Tessier-Lavigne M, Goodman CS, Dickson BJ (1996). Genetic analysis of netrin genes in Drosophila: netrins guide CNS commissural axons and peripheral motor axons. Neuron.

[41] O’Connor-Giles KM, Ganetzky B (2008). Satellite signaling at synapses. Fly.

[42] O’Connor-Giles KM, Ho LL, Ganetzky B (2008). Nervous wreck interacts with thickveins and the endocytic machinery to attenuate retrograde BMP signaling during synaptic growth. Neuron.

[43] Özkan E, Carrillo RA, Eastman CL, Weiszmann R, Waghray D, Johnson KG, Zinn K, Celniker SE, Garcia KC (2013). An extracellular interactome of immunoglobulin and LRR proteins reveals receptor-ligand networks. Cell.

[44] Pappu KS, Morey M, Nern A, Spitzweck B, Dickson BJ, Zipursky SL (2011). Robo-3–mediated repulsive interactions guide R8 axons during Drosophila visual system development. Proc National Acad Sci.

[45] Pischedda F, Piccoli G (2016). The IgLON family member Negr1 promotes neuronal Arborization acting as soluble factor via FGFR2. Front Mol Neurosci.

[46] Poovathumkadavil P, Jagla K (2020). Genetic control of muscle diversification and homeostasis: insights from Drosophila. Cells.

[47] Ranganayakulu G, Elliott DA, Harvey RP, Olson EN (1998). Divergent roles for NK-2 class homeobox genes in cardiogenesis in flies and mice. Development.

[48] Rougon G, Hobert O (2003). New insights into the diversity and function of neuronal immunoglobulin superfamily molecules. Annu Rev Neurosci.

[49] Schindelin J, Arganda-Carreras I, Frise E, Kaynig V, Longair M, Pietzsch T, Preibisch S, Rueden C, Saalfeld S, Schmid B, Tinevez J-Y, White DJ, Hartenstein V, Eliceiri K, Tomancak P, Cardona A (2012). Fiji: an open-source platform for biological-image analysis. Nat Methods.

[50] Sehadova H, Glaser FT, Gentile C, Simoni A, Giesecke A, Albert JT, Stanewsky R (2009). Temperature entrainment of Drosophila’s circadian clock involves the gene nocte and signaling from peripheral sensory tissues to the brain. Neuron.

[51] Sen A, Yokokura T, Kankel MW, Dimlich DN, Manent J, Sanyal S, Artavanis-Tsakonas S (2011). Modeling spinal muscular atrophy in Drosophila links Smn to FGF signaling. J Cell Biol.

[52] Shimozono M, Osaka J, Kato Y, Araki T, Kawamura H, Takechi H, Hakeda-Suzuki S, Suzuki T (2019). Cell surface molecule, Klingon, mediates the refinement of synaptic specificity in the Drosophila visual system. Genes Cells.

[53] Singh K, Lilleväli K, Gilbert SF, Bregin A, Narvik J, Jayaram M, Rahi M, Innos J, Kaasik A, Vasar E, Philips MA (2018). The combined impact of IgLON family proteins Lsamp and Neurotrimin on developing neurons and behavioral profiles in mouse. Brain Res Bull.

[54] Stoeckli ET (2018). Understanding axon guidance: are we nearly there yet?. Development.

[55] Venkatasubramanian L, Guo Z, Xu S, Tan L, Xiao Q, Nagarkar-Jaiswal S, Mann RS (2019). Stereotyped terminal axon branching of leg motor neurons mediated by igsf proteins dip-α and dpr10. Elife.

[56] Wang Y, Lobb-Rabe M, Ashley J, Chatterjee P, Anand V, Bellen HJ, et al. Systematic expression profiling of dprs and DIPs reveals cell surface codes in Drosophila larval peripheral neurons. Biorxiv. 2021. 10.1101/2021.10.20.465173. 10.1242/dev.200355 PMC9188756 35502740

[57] Winberg ML, Noordermeer JN, Tamagnone L, Comoglio PM, Spriggs MK, Tessier-Lavigne M, Goodman CS (1998). Plexin a is a neuronal Semaphorin receptor that controls axon guidance. Cell.

[58] Wu H, Xiong WC, Mei L (2010). To build a synapse: signaling pathways in neuromuscular junction assembly. Development.

[59] Wu R, Li A, Sun B, Sun J-G, Zhang J, Zhang T, Chen Y, Xiao Y, Gao Y, Zhang Q, Ma J, Yang X, Liao Y, Lai W-Y, Qi X, Wang S, Shu Y, Wang H-L, Wang F, Yang Y-G, Yuan Z (2019). A novel m6A reader Prrc2a controls oligodendroglial specification and myelination. Cell Res.

[60] Xu C, Theisen E, Maloney R, Peng J, Santiago I, Yapp C, Werkhoven Z, Rumbaut E, Shum B, Tarnogorska D, Borycz J, Tan L, Courgeon M, Meinertzhagen IA, Bivort B d, Drugowitsch J, Pecot MY (2019). Control of synaptic specificity by establishing a relative preference for synaptic partners. Neuron.

[61] Xu S, Xiao Q, Cosmanescu F, Sergeeva AP, Yoo J, Lin Y, Katsamba PS, Ahlsen G, Kaufman J, Linaval NT, Lee P-T, Bellen HJ, Shapiro L, Honig B, Tan L, Zipursky SL (2018). Interactions between the Ig-superfamily proteins DIP-α and Dpr6/10 regulate assembly of neural circuits. Neuron.

[62] Yoshihara M, Rheuben MB, Kidokoro Y (1997). Transition from growth cone to functional motor nerve terminal in Drosophila embryos. J Neurosci.

