# Supplementary figures and images for: Dpr10 and Nocte are required for Drosophila motor axon pathfinding
Source: Neural Dev. 2022 Oct 21;17:10. doi: 10.1186/s13064-022-00165-5 (PMC9585758; doi:10.1186/s13064-022-00165-5)

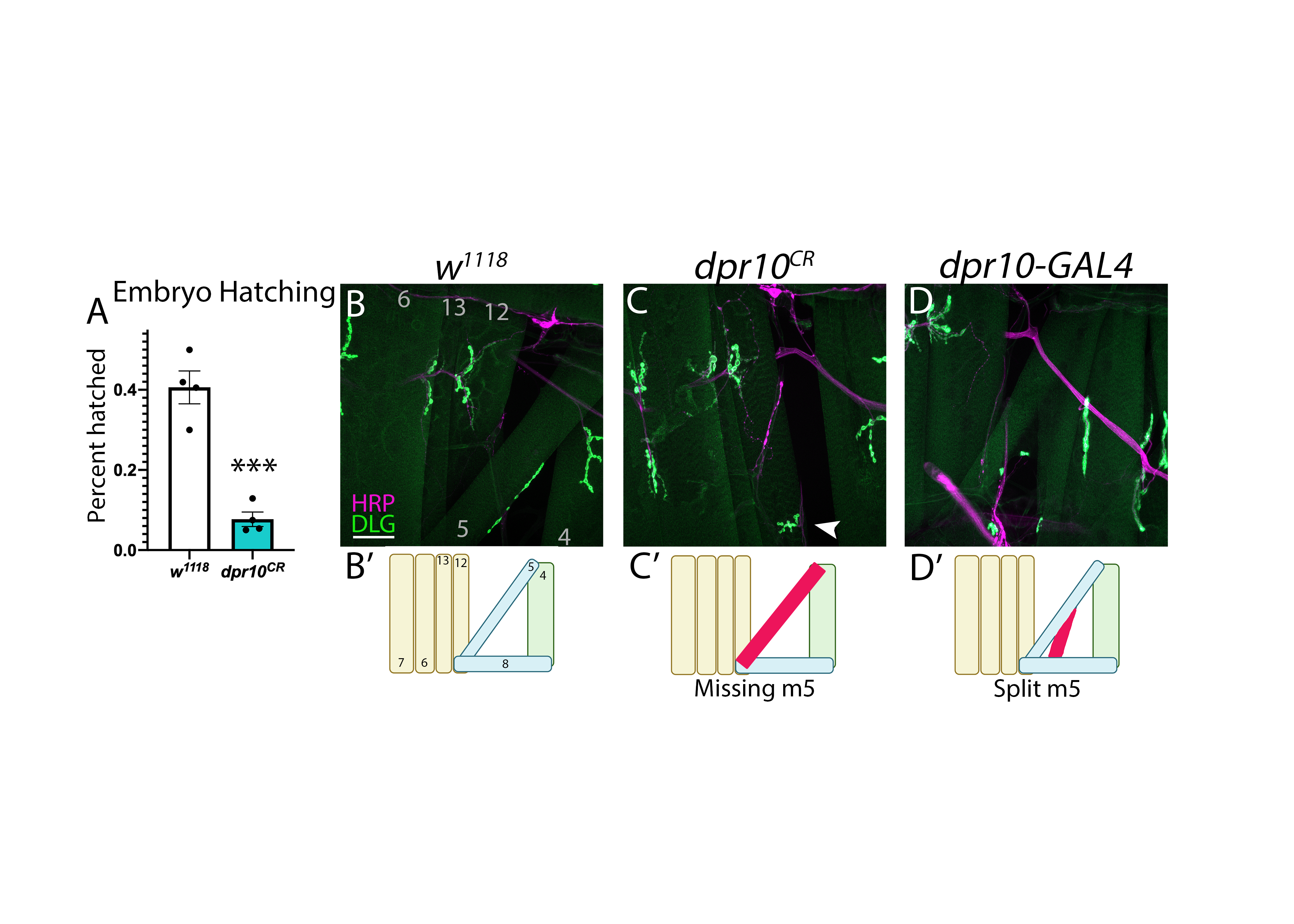

Supplement: Supplementary file 1 — Additional file 1: Supplementary Fig. 1 (Supplement to Fig. 1): Embryo hatching and muscle patterning defects in dpr10 mutants. (A) Percentage of hatched embryos in w1118 and dpr10CR backgrounds, ***p < 0.001. (B) A control w1118 animal depicting the normal muscle patterns in the ventral field. Neurons were labeled by staining for HRP (magenta) and postsynapses were labeled by staining for DLG (green). Note that the GFP channel also outlines individual muscles. (C) A dpr10CR animal with a missing m5. Ectopic innervation of m12 by MN5-Ib indicated with arrowhead. (D) A dpr10-GAL4 animal with a split m5. (B′-D′) Cartoon schematics of muscle patterning observed in A-C. Aberrant muscles highlighted in red. Scale bar = 50 μm. [file 13064_2022_165_MOESM1_ESM.tif]

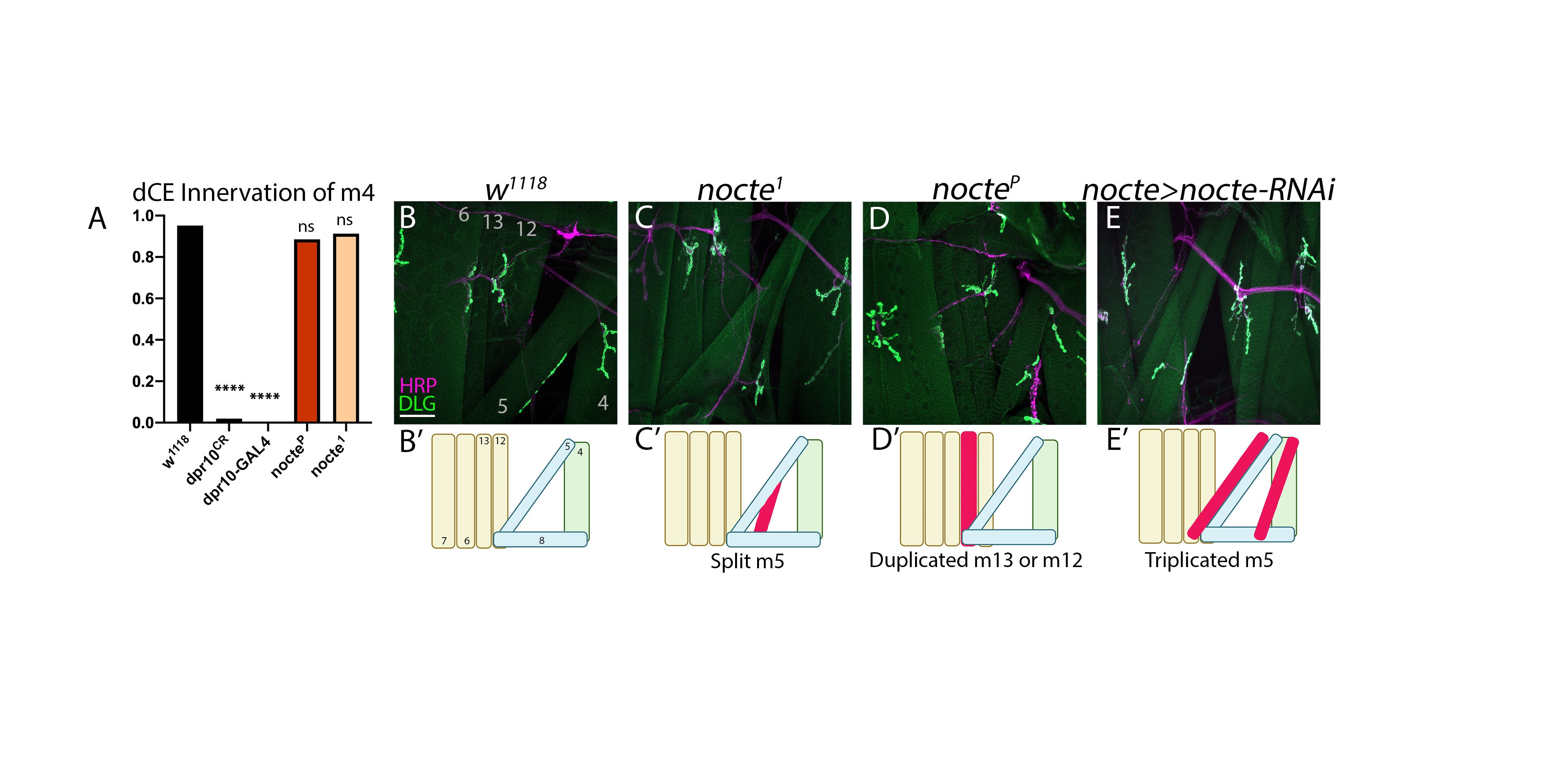

Supplement: Supplementary file 2 — Additional file 2: Supplementary Fig. 2 (Supplement to Fig. 3): nocte mutants exhibit normal innervation of m4 but display muscle defects. (A) Innervation frequency of m4 by the dCE (Is neuron that innervates the dorsal muscles) in respective genotypes. Loss of nocte does not affect dCE innervation of m4. ****p < 0.0001. (B) A control w1118 animal with normal muscle patterns. Neurons were labeled by staining for HRP (magenta) and postsynapses were labeled by staining for DLG (green). (C) A nocte1 animal with a split m5. (D) A nocteP animal with duplicated m13 or m12. (E) A nocte-GAL4 x nocte-RNAi animal with a triplicated m5. (B′-E′) Cartoon schematics of muscle patterns observed in B-E. Aberrant muscles shown in red. Scale bar = 50 μm. [file 13064_2022_165_MOESM2_ESM.tif]

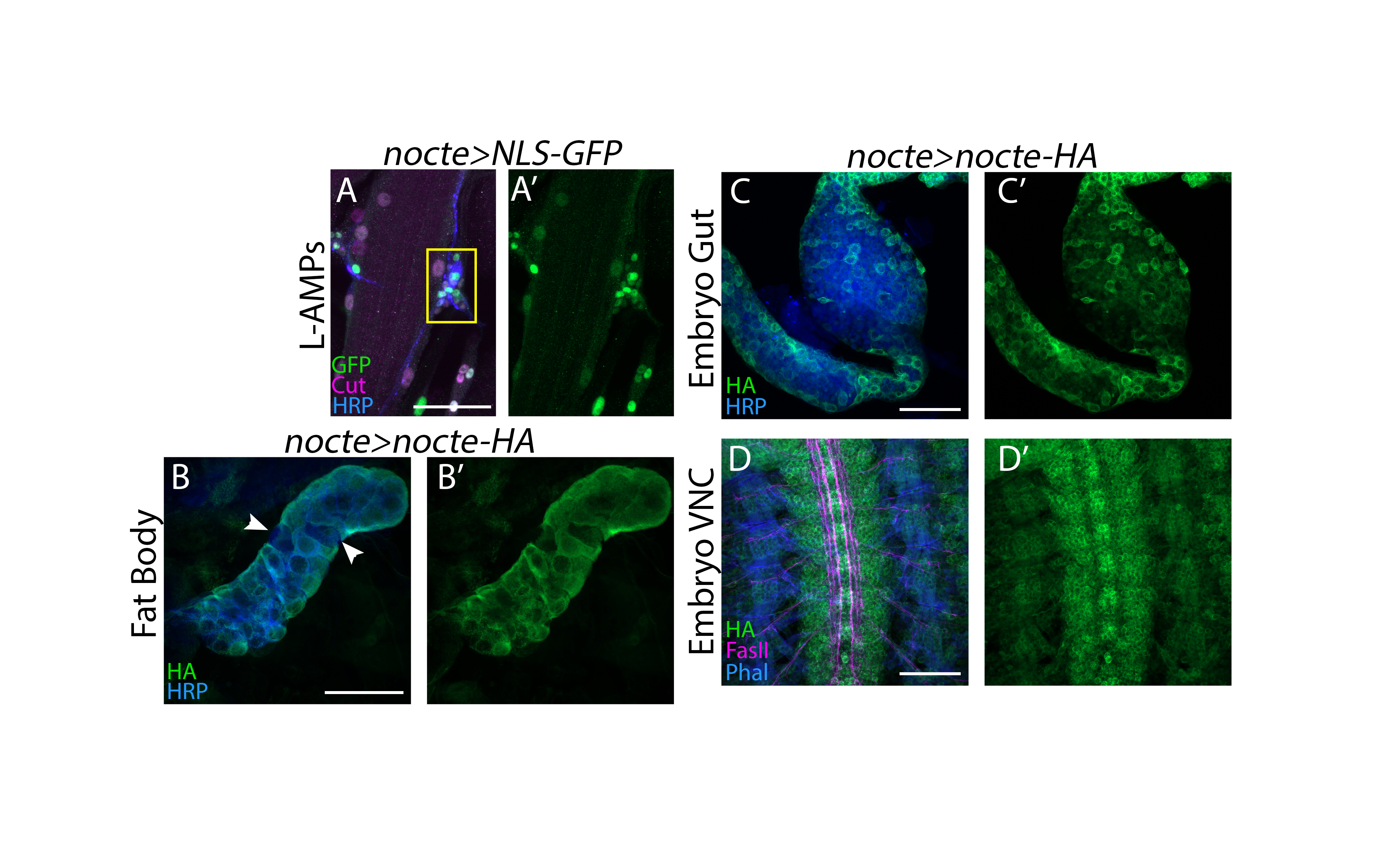

Supplement: Supplementary file 3 — Additional file 3: Supplementary Fig. 3 (Supplement to Fig. 5): Nocte peripheral expression. (A) nocte-GAL4 x UAS-NLS-GFP larvae. Lateral adult muscle precursor cells (L-AMPs) highlighted by a yellow rectangle. Preps were co-stained for the transcription factor Cut (magenta) and HRP (blue). (A′) GFP channel. (B-D) nocte-GAL4 x UAS-nocte-HA larvae. (B) Larval fat body cells. Nocte localization labeled with HA (green) and FB outline shown in HRP (blue). Cells lacking nocte expression indicated with arrowhead. (C) Embryonic gut dissected away from rest of embryo. Nocte localization labeled with HA (green) and gut outline shown in HRP (blue). (D) In the embryonic VNC, transgenic Nocte expression visualized with HA (green), nerves labeled with FasII (magenta), and muscles labeled with phalloidin (blue). All scale bars = 50 μm. [file 13064_2022_165_MOESM3_ESM.tif]

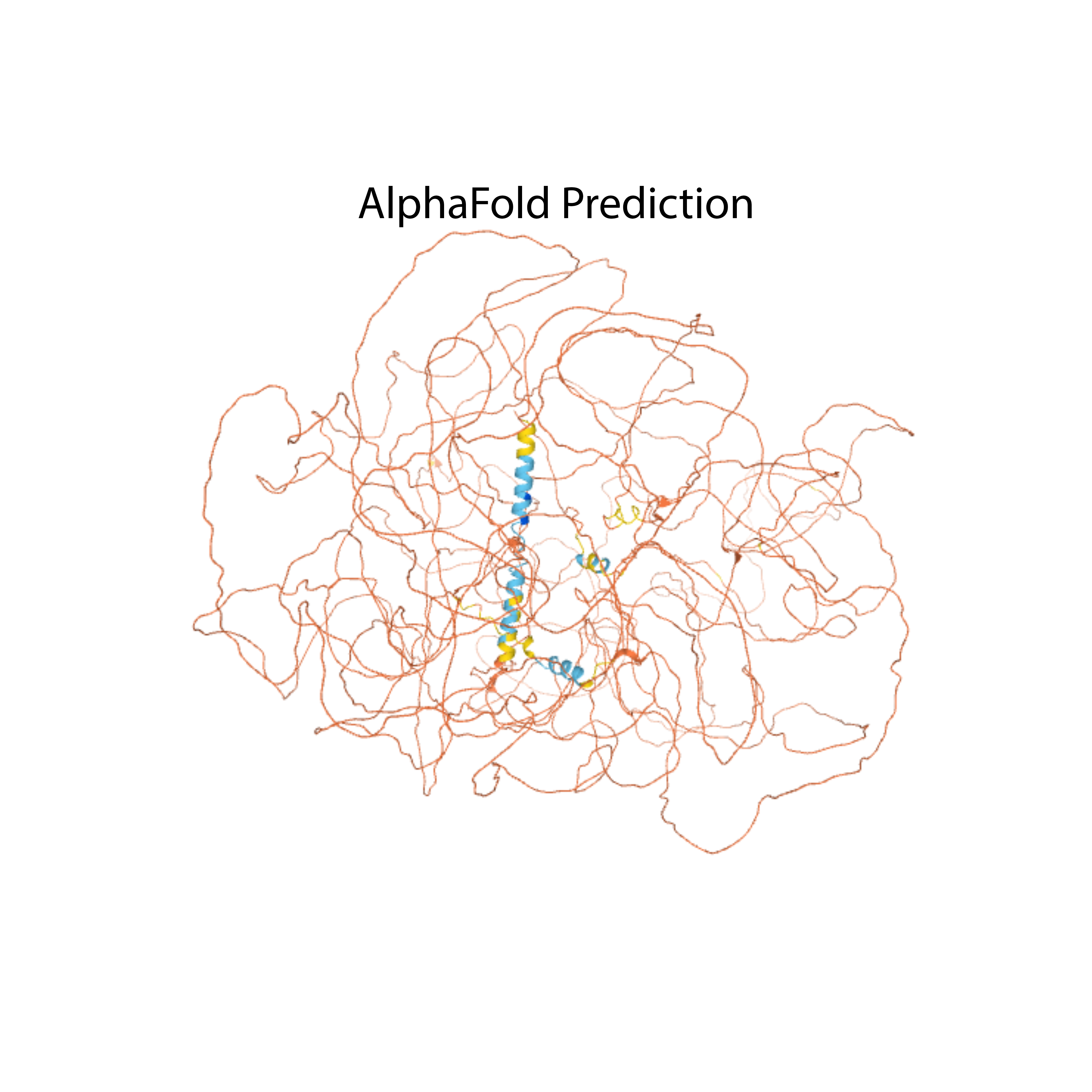

Supplement: Supplementary file 4 — Additional file 4: Supplementary Fig. 4 (Supplement to Fig. 6): AlphaFold structural prediction of Nocte. The orange lines are predicted unstructured regions with fragments of alpha helices visible in the center. [file 13064_2022_165_MOESM4_ESM.tif]

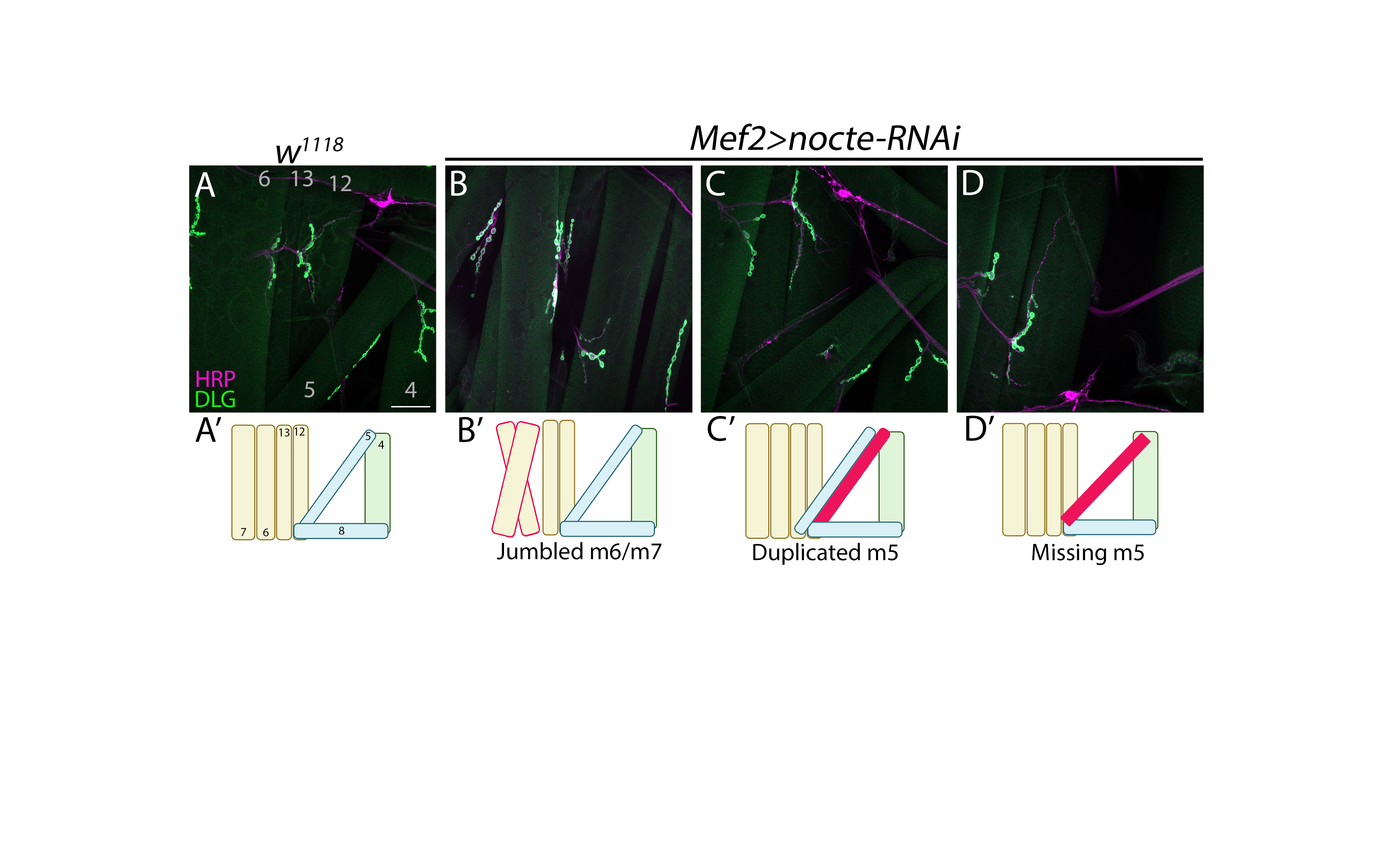

Supplement: Supplementary file 5 — Additional file 5: Supplementary Fig. 5 (Supplement to Fig. 7): Muscle patterning defects caused by knockdown of nocte in muscles. (A) A control w1118 animal depicting the normal muscle pattern. Neurons are labeled by HRP staining (magenta) and postsynapses by DLG staining (green). Note that the outline of muscles can be clearly visualized in the green channel. Scale bar = 50 μm. (B-D) Mef2-GAL4 x UAS-nocte-RNAix2 animals revealed several defects including (B) crisscrossing of m6 and m7, (C) duplication of m5, and (D) absence of m5. (A′-D′) Cartoon schematics of muscle patterns observed in A-D. Aberrant muscles shown in red. Scale bar = 50 μm. [file 13064_2022_165_MOESM5_ESM.tif]

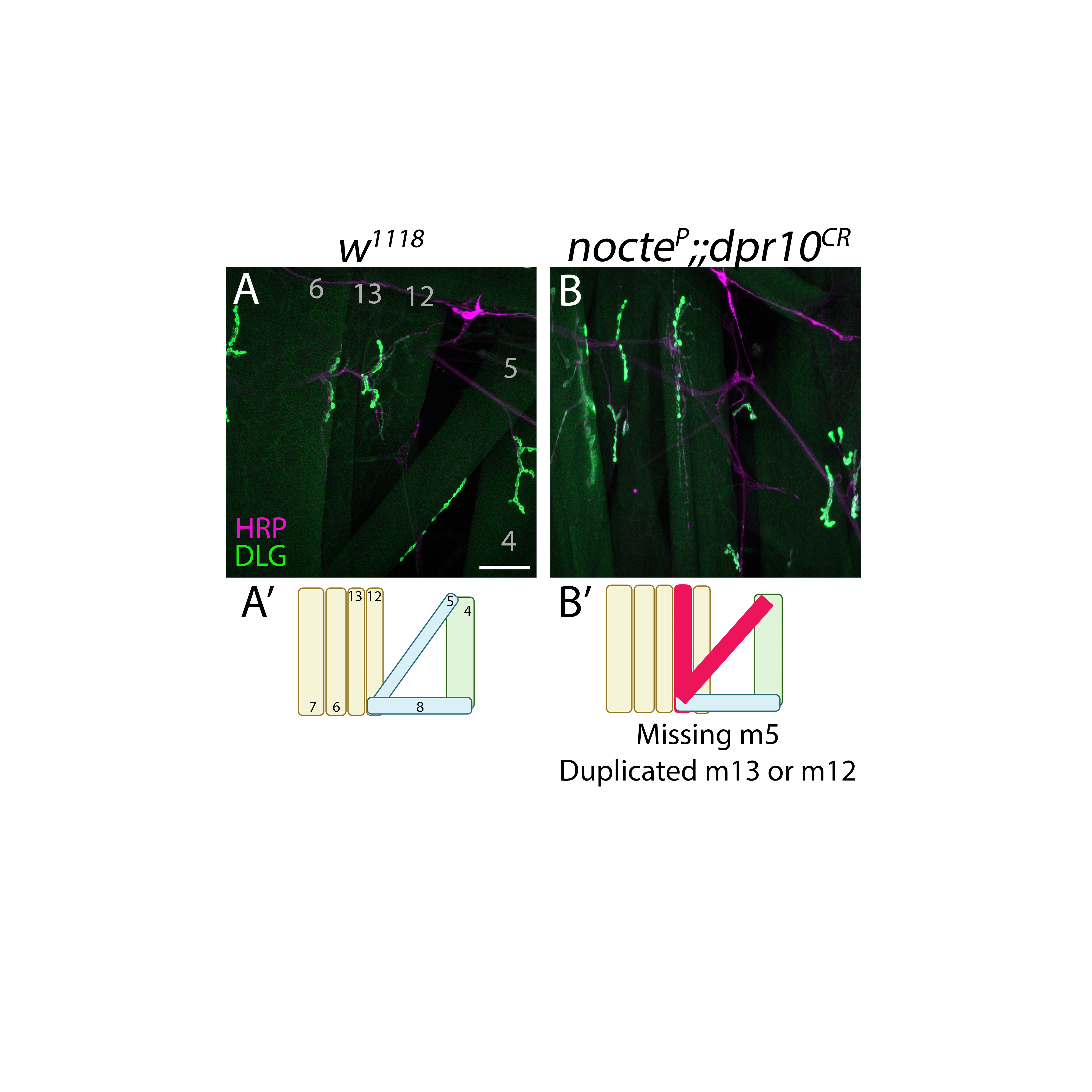

Supplement: Supplementary file 6 — Additional file 6: Supplementary Fig. 6 (Supplement to Fig. 8): Muscle defects in nocte,dpr10 double mutants. (A) A control w1118 animal depicting the normal muscle pattern. Neurons are labeled by HRP staining (magenta) and postsynapses by DLG staining (green). Note that the outline of muscles can be clearly visualized in the green channel. Scale bar = 50 μm. (B) nocteP;;dpr10CR double mutant animals showed various muscle patterning defects including duplication of m13 or m12 and missing m5. (A′-B′) Cartoon schematics of muscle patterns observed in A-B. Aberrant muscles shown in red. Scale bar = 50 μm. [file 13064_2022_165_MOESM6_ESM.tif]
